# Unravelling the Molecular Responses of the Yeast *Schwanniomyces etchellsii* to Hyperosmotic Stress in Seawater Medium Using Omic Approaches

**DOI:** 10.3390/ijms27010183

**Published:** 2025-12-23

**Authors:** Cecilia Andreu, Èlia Obis, Marcel·lí del Olmo

**Affiliations:** 1 Departament de Química Orgànica, Facultat de Farmàcia, Universitat de València, E-46100 Burjassot, València, Spain; 2 Department of Experimental Medicine, University of Lleida-Lleida Biomedical Research Institute (UdL-IRBLleida), E-25198 Lleida, Spain; elia.obis@udl.cat; 3 Departament de Bioquímica i Biologia Molecular, Facultat de Ciències Biològiques, Universitat de València, E-46100 Burjassot, València, Spain

**Keywords:** Ena1, ergosterol, glycerol-3-phosphate dehydrogenase, lipidomics, osmotic stress response, proteomics, *Schwanniomyces etchellsii*, seawater

## Abstract

*Schwanniomyces etchellsii* is an unconventional, halotolerant microorganism. Like some other yeasts, it can efficiently perform various biocatalytic transformations of organic compounds in seawater more effectively than in freshwater. In seawater, conversion rates are higher, by-product production is minimized, greater substrate loading is possible, and cells can be recycled for further use. To identify the molecular features that explain this behavior, comparative proteomic and lipidomic studies were conducted on cells grown in seawater and freshwater at various growth stages. The results showed higher expression of proteins involved in the stress response, such as glycerol-3-phosphate dehydrogenase, the glycerol transporter Stl1 and the P-type ATPase sodium pump Ena1, and several phospholipid biosynthesis proteins, including inositol-3-phosphate synthase and phosphatidate cytidylyltransferase, in seawater. Changes in metabolic enzymes and other proteins involved in responding to stimuli were also observed between the two conditions. Overall, cells grown in a freshwater medium exhibited higher levels of enzymes involved in biosynthetic processes. Differences in lipid profiles were also observed between cells grown in the two media. Higher levels of monoacyl and diacylglycerols were found in seawater, while higher levels of phospholipids containing serine and ethanolamine were found in freshwater. Consistent with more permeable membranes, cells grown in seawater exhibited lower levels of ergosterol.

## 1. Introduction

*Schwanniomyces etchellsii* is an unusual yeast strain that has been found in fermented vegetables [1,2] and in the Lombok seawater area [3]. It belongs to the Debaryomycetaceae family. Although it was previously included in the *Debaryomyces* or *Pichia* genus, studies by Kurtzman and Suzuki reassigned it to the *Schwanniomyces* genus, along with *S.* (*D*.) *polymorphus* [4]. Previous studies have verified that this microorganism can grow in the presence of NaCl concentrations of at least 7.5% (*w*/*v*), and that it exhibits different behavior depending on the salinity of the medium [5].

In our laboratory, we use *S. etchellsii* for the stereoselective synthesis of compounds of pharmaceutical and chemical interest. We sometimes use seawater as a solvent for this purpose. We have demonstrated that this microorganism can produce (*R*)-phenylacetylcarbinol (PAC), a chiral precursor to the drug ephedrine, with higher yields and fewer byproducts in seawater than in freshwater [5]. Reduction reactions of several prochiral ketones were also carried out more effectively in this medium [6,7]. Further studies have shown that *S. etchellsii* is more resistant to organic solvents and compounds in seawater than in freshwater. This allows for an increased load of the starting material. Metabolic activity is maintained in seawater, enabling better performance in subsequent recycling steps for several rounds and reducing industrial costs. Additionally, previous studies have demonstrated that *S. etchellsii* exhibits higher thermotolerance in seawater media [6,7]. The behavior of this yeast as a biocatalyst in seawater makes it particularly interesting for biotechnological processes that use large amounts of water, underscoring the importance of further researching its properties.

Another interesting characteristic of *S. etchellsii* in seawater that is not exhibited in freshwater is its ability to form biofilms in nutrient-rich environments without a carbon source. These biofilms can form on silicone surfaces and at air-liquid interfaces. The extracellular matrix of these communities consists primarily of carbohydrates and enzymes involved in biosynthetic processes, including glyceraldehyde-3-phosphate dehydrogenase and ATP synthase subunit β, as well as proteins related to the response to stimuli, such as those involved in protein folding [8]. To determine the molecular mechanisms that could explain this yeast’s ability to form such structures, the proteome of biofilm cells embedded in the extracellular matrix was compared with the proteome of planktonic or non-biofilm-forming cells. In both cases, the biofilm cells contained proteins overexpressed and involved in protein biosynthesis, membrane structure, and vesicle-mediated transport. The large number of proteins involved in translation and located in ribosomes suggests that they play a greater role in protein biosynthesis than their counterparts. These analyses revealed a homolog of *Candida albicans* Spf1p in *S. etchellsii*. This P-type ATPase ion transporter is involved in several processes in *C. albicans*, including cell adhesion, cell wall organization, and biogenesis [9].

No other molecular analyses of *S. etchellsii* have been previously published, and its genome has not yet been sequenced. The closest related yeast for which such information is available is *Debaryomyces hansenii*. Several studies have characterized *D. hansenii* genome sequences [10,11] or provided insights into its molecular adaptations to hypersaline stress [12,13,14,15,16,17,18,19]. One of these studies provided a proteomic description of changes due to modified external osmolarity at 8% and 12% (*w*/*v*) NaCl [15], and another one reported phosphoproteomic and proteomic profiles in the presence of 1 M NaCl or KCl [19]. Along with the aforementioned studies, the only other proteomic studies conducted thus far on this yeast, to our knowledge, are those investigating the response of *D. hansenii* to potassium starvation or perchlorate stress by Martínez et al. and Heinz et al., respectively [20,21].

In the present study, we employ omic approaches to elucidate the molecular mechanisms underlying *S. etchellsii*’s adaptation to seawater and its enhanced chemical reaction efficiency in this medium. Proteomic and lipidomic analyses revealed an increased expression of a protein homolog of the *S. cerevisiae* P-type ATPase sodium pump Ena1 in seawater, as well as other proteins found in other yeasts that are involved in the response to osmotic stress. Additionally, we observed variations in biosynthetic activity, as well as differences in the levels of proteins associated with phospholipid biosynthesis and in the quantities of monoacyl- and diacylglycerols, and some phospholipids, depending on the composition of the culture medium.

## 2. Results

### 2.1. The Kinetics of S. etchellsii Growth in YPD and SW-YPD

In previous work, we confirmed that *S. etchelsii* exhibits enhanced catalytic activity in organic reactions carried out in seawater compared to identical conditions in a freshwater medium [5,6,7]. We also observed increased resistance to organic solvents, a higher substrate loading capacity, and greater thermostability in the former medium. To better understand the molecular basis of these behaviors, we performed comparative proteomic and lipid analyses on samples obtained after yeast growth in both media.

**Figure 1:**
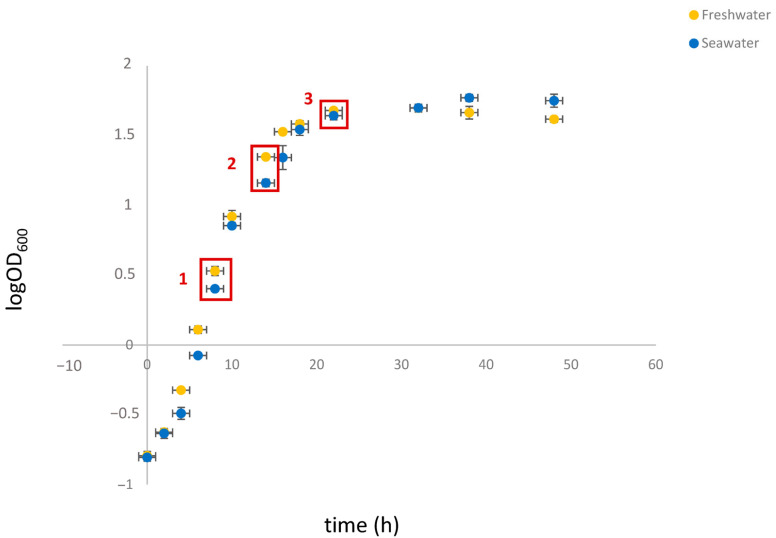
Growth curves of *S. etchellsii* in YPD and SW-YPD. Cells from overnight cultures in YPD or SW-YPD were transferred to the corresponding medium at an OD_600_ of 0.3, after which growth continued for two days. Red squares indicate the time points selected for proteomic analysis. Experiments were carried out in triplicate, and the data shown corresponds to the mean and standard deviation.

To this end, cell extracts were prepared at significant stages throughout the growth cycle. To determine the appropriate time points, the growth kinetics of *S. etchellsii* in YPD and seawater-YPD (SW-YPD) were examined. As can be seen in Figure 1, a growth delay was observed in the seawater medium for at least the first 20 h. However, when the cells reached the stationary phase, the optical density at 600 nm (OD_600_) decreased more rapidly in the freshwater medium. Analysis of these growth curves determined the optimal time points for comparing protein expression in both media: 8, 14, and 22 h after culture inoculation. These time points correspond to the log, mid-log, and late log phases of growth, with OD_600_ values of around 2–3, 15–20, and 45–50, respectively.

In addition, we were interested in determining the effect of a sudden change from a freshwater to a seawater environment. To this end, we compared cell extracts obtained from *S. etchellsii* cultures in YPD and SW-YPD, which were prepared by transferring cells from a previous YPD culture, as is described in the Section 4.

### 2.2. Proteomic Analyses of S. etchellsii Samples Obtained at Various Stages of the Growth Curve

Table 1 summarizes the overall results of the proteomic comparison between SW-YPD and YPD after False Discovery Rate (FDR) filtering (<1%). More than a quarter of the total yeast proteins were identified in each experiment. Analyzing the samples corresponding to the time points of the growth curves revealed significant differences. Approximately 300 proteins exhibited differential levels during log-phase growth (run 1 in Table 1), with a similar distribution of up- and down-regulated proteins in seawater. Conversely, more than 400 proteins exhibited significant changes in quantity during mid-log phase (run 2), with a higher proportion of up-regulated proteins in freshwater. There were few differences in protein expression between the two media in the late log phase (run 3). Finally, in the extracts obtained after the sudden shift to seawater (run 4), approximately 250 proteins were differentially expressed, with a higher percentage of them found to be up-regulated in seawater. This table shows data about proteins with differential expression levels greater than 1.3 between the considered conditions. For future analyses, however, we will consider only those with variations greater than 1.5.

**Figure 2:**
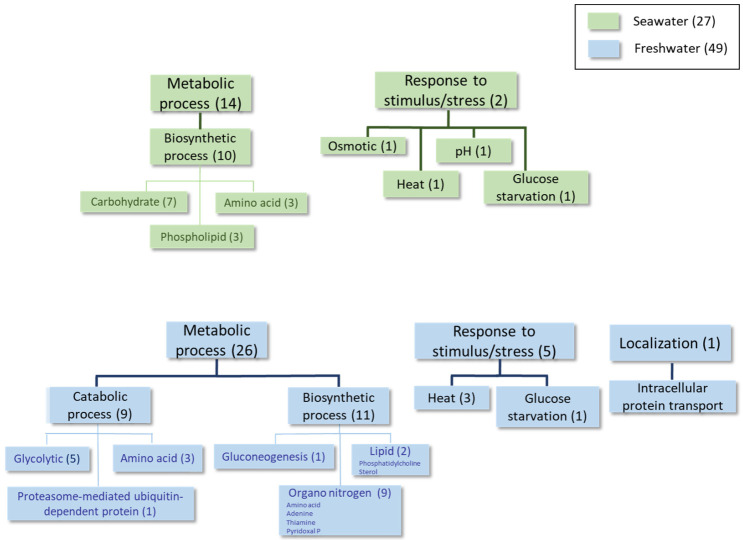
Classification of proteins differentially expressed at least 1.5-fold in condition 1 according to GO categories of Biological Process. Categories that are overexpressed in seawater are shaded green, and categories that are overexpressed in freshwater are shaded blue.

**Figure 3:**
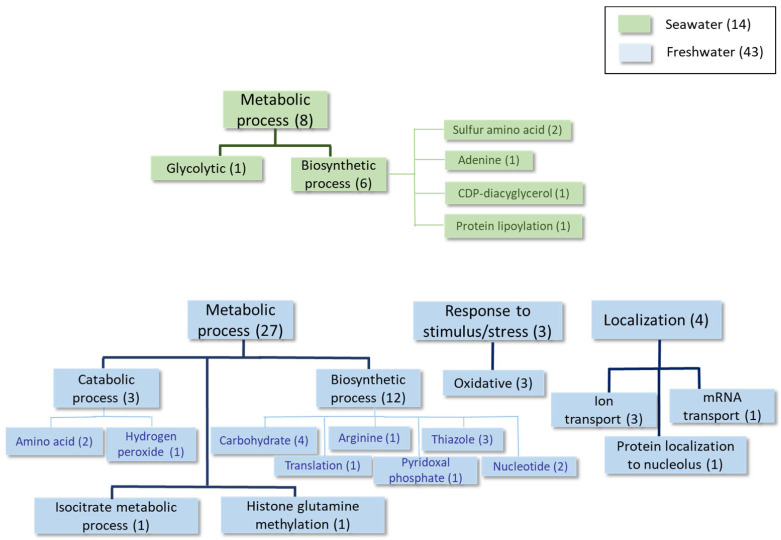
Classification of the proteins differentially expressed at least 1.5-fold in condition 2 according to the GO categories of Biological Process.

**Figure 4:**
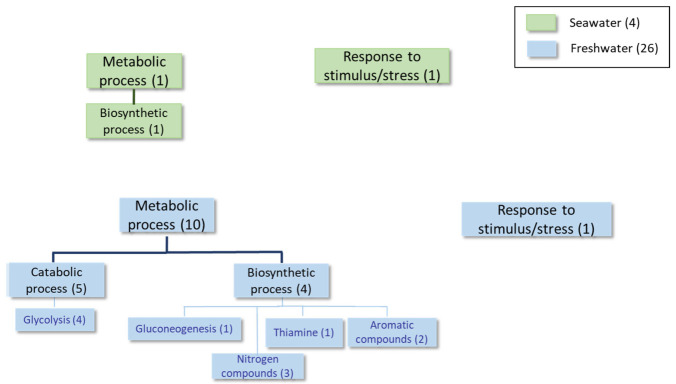
Classification of the proteins differentially expressed at least 1.5-fold in condition 3 according to the GO categories of Biological Process.

**Figure 5:**
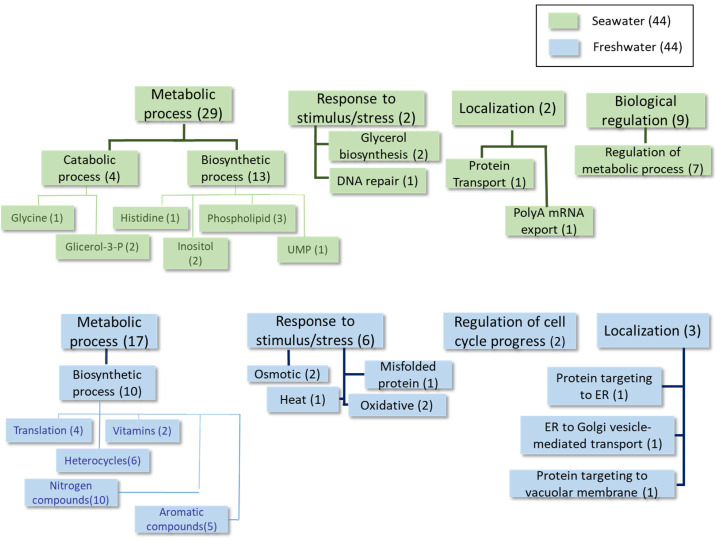
Classification of the proteins differentially expressed at least 1.5-fold in condition 4 according to the GO categories of Biological Process.

The detailed results obtained using Universal Protein Resources (UniProt (Release 2025_01) tools [22]) at each time point of the growth curve are shown in Table 2, Table 3, Table 4, Table 5, Table 6, Table 7, Table 8 and Table 9 and Figure 2, Figure 3, Figure 4 and Figure 5, as well as Tables S2–S5 in the Supplementary Materials. For simplicity, Table 2, Table 4 and Table 6 list the proteins expressed at least twofold higher in seawater than in the freshwater medium during the log, mid-log, and late log phases of growth, respectively. Those showing higher levels in freshwater than in seawater at the same time points and cutoff are shown in Table 3, Table 5 and Table 7. The proteomic analysis following a sudden change from a freshwater to a seawater medium is shown in Table 8 and Table 9. Figure 2, Figure 3, Figure 4 and Figure 5 and Tables S2–S5 summarize information about proteins with fold-change values of at least 1.5 under each condition.

In the analysis performed during the **early log phase** (see Table 2 and Table 3, Figure 2 and Supplementary Table S2), homologous proteins to the *S. cerevisiae* transporters Ena1 (a P-type ATPase sodium pump) and Stl1 (a glycerol-proton symporter) were identified as being overexpressed in the seawater medium (see the shaded rows in Table 2). These proteins are both up-regulated by osmotic shock in this yeast [23,24]. Some biosynthetic proteins involved in phospholipid and amino acid metabolism were also found under these conditions. Conversely, proteins belonging to the categories of catabolic and biosynthetic processes (primarily organonitrogen compounds, such as thiamine, and several amino acids), as well as some stress response proteins, were identified as overexpressed in the freshwater medium at this time point.

Similarly to the above-described data, higher expression of the Ena1 homolog (shaded in Table 4) was found during the **mid-logarithmic growth phase** in a seawater medium. This was also observed for proteins related to the response to oxidative stress and phospholipid biosynthesis (Table 4, Figure 3 and Supplementary Table S3). This is the case for phosphatidate cytidylyltransferase, which synthesizes cytidine diphosphate diacylglycerol (CDP-DAG). CDP-DAG is a key intermediate in the generation of phosphatidylinositol and cardiolipin [25]. In freshwater, proteins mainly associated with carbohydrate, nucleotide, and thiazole biosynthesis were detected as being upregulated at this time point, together with other proteins involved in the response to oxidative stress and ion transport (Table 5, Figure 3 and Supplementary Table S3).

In the **advanced log phase**, few proteins with different expression levels in both media were found. However, the presence of the Ena1 homolog, which was up-expressed in the seawater medium (shaded rows in Table 6), is relevant. Several enzymes involved in both biosynthetic and catabolic metabolic processes were up-regulated in the freshwater medium (see Table 6 and Table 7, Figure 4 and Supplementary Table S4). Notably, several proteins related to carbohydrate metabolism and thiamine biosynthesis were identified.

Finally, increased levels of glycerol-3-phosphate dehydrogenase were found in the extracts obtained after the cells experienced **osmotic shock** from being transferred from a freshwater environment to a seawater environment (see shaded row in Table 8, Figure 5 and Supplementary Table S5). This enzyme is associated with glycerol production, which counteracts the increase in osmolarity [26]. The detection of pyruvate carboxylase is also notable. It is involved in gluconeogenesis and lipogenesis, and it appeared at higher levels in seawater in other comparisons carried out in this study (see Tables S2 and S3 in the Supplementary Materials). The other proteins identified as differentially up-regulated in the same media corresponded to enzymes involved in several metabolic pathways, primarily those involved in inositol, phospholipid, histidine, and uridine monophosphate synthesis. Regarding the proteins overexpressed in freshwater (Table 9, Figure 5 and Supplementary Table S5), several proteins involved in biosynthetic processes were identified, particularly those related to nitrogen compounds (e.g., nucleotides, amino acids, and vitamins), as well as proteins involved in cellular division, protein folding, and targeting. Several stress response proteins were also present.

**Figure 6:**
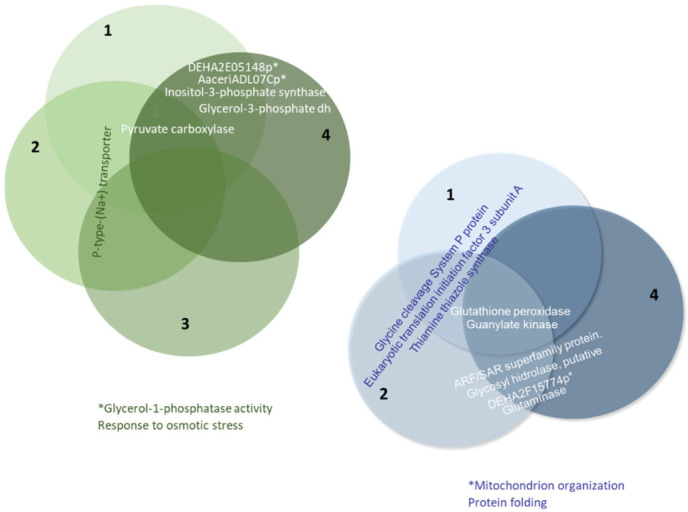
Proteins with differential expression levels that are at least 1.5-fold higher under more than one of the conditions considered in this study.

Considering all proteins with differential expression levels of at least 1.5-fold across the four conditions studied in this work, we found that some proteins were up-regulated in the same medium in several cases (see Figure 6 and Table S6 in the Supplementary Materials). For example, pyruvate carboxylase enzymes were overexpressed in the seawater medium under conditions 1, 2, and 4. A similar pattern was observed with the P-type Na^+^ transporter under conditions 1, 2, and 3. Two conditions (1 and 4) involved proteins in lipid biosynthesis (e.g., inositol-3-phosphate synthase) and glycerol production for the osmotic stress response (e.g., glycerol-1-phosphatase and glycerol-3-phosphate dehydrogenase). Conversely, three conditions (1, 2, and 4) exhibited higher expression of guanylate kinase and glutathione peroxidase enzymes in the freshwater medium. Thiamine, thiazole synthase, glycine cleavage system P protein, and eukaryotic translation initiation factor 3 subunit A were detected in conditions 1 and 2. Glutaminase, DHA2F15774p (a protein involved in protein folding and mitochondrial organization), a putative glycoside hydrolase, and a protein of the ARF/SAR superfamily (ADP-ribosylation factor) were present in conditions 2 and 4.

### 2.3. Sequence Alignment Between S. cerevisiae Ena1 Homologs in Unconventional Yeasts

Unfortunately, the *S. etchellsii* genome has not yet been sequenced, so many of the proteins described in this study were identified based on their homology with proteins from other yeasts. Of the differentially up-expressed proteins in seawater (Table 2, Table 4, Table 6 and Table 8), most have high similarity with their counterparts in *D. hansenii*, *M. guilliermondii*, and *S. cerevisiae* (Table S7), typically showing higher similarity with the first two yeasts.

**Figure 7:**
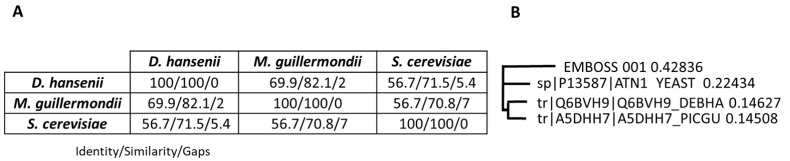
Alignments of yeast Ena1 proteins. (**A**) Data from the pairwise alignments using the EMBOSS Matcher facility [28]. (**B**) Deduced phylogenetic tree obtained by Clustal Omega Multiple Sequence Alignment [29]. EMBOSS_001 refers to the *S. etchellsii* protein identified in this study, YEAST to *S. cerevisiae*, DEBHA to *D. hansenii* and PICGU to *M. guilliermondii*.

One such protein is Ena1. The proteomic studies described in this work identified a homolog of Ena1-like proteins in *M. guilliermondii* and *D. hansenii*. This prompted us to compare the sequences of these proteins with the *S. cerevisiae* Ena1 protein using the Basic Local Alignment Search Tool (BLAST + 2.17.0 [27]) (see Supplementary Figure S1). This analysis revealed the presence of an N-terminal sequence containing 27 amino acids in the *D. hansenii* protein and 19 amino acids in the *M. guilliermondii* protein. The D. *hansenii* homolog shows 71.5% similarity to the *S. cerevisiae* Ena1 sequence, while the *M. guilliermondii* homolog shows 70.8% similarity (Figure 7). The *D. hansenii* and *M. guilliermondii* homologs showed 82.1% similarity. These results suggest that the proteins of the two non-*Saccharomyces* yeasts are more closely related to each other than to the *S. cerevisiae* protein (Figure 7). Supplementary Figure S1 also includes the peptide sequences found in this study that allowed identification of an Ena1 homolog in *S. etchellsii*. Unfortunately, the available sequence does not allow us to infer the phylogenetic relationship with the other proteins.

### 2.4. Lipidomics Analyses

Proteomic analyses thus far have revealed differences in the expression of certain proteins involved in lipid biosynthesis between cells grown in seawater and freshwater. To determine if these differences result in changes in specific lipid levels under both conditions, non-targeted lipidomics analyses were performed using univariate and multivariate statistical approaches. We employed Principal Component Analysis (PCA), an unsupervised multivariate method, to reduce dimensionality of the data and explore natural clustering among samples based on global lipidomic variation [30]. PCA enabled visualization of group separation and identification of potential outliers.

Random Forest, a supervised machine learning algorithm, was then applied to further classify samples and highlight important lipid species contributing to group differences. This method ranked lipids according to their importance in discriminating between conditions, providing insight into key biomarkers [31]. Additionally, hierarchical clustering and heatmaps were generated to visualize the relative abundance of significantly altered lipid species and detect co-regulated lipid patterns across samples.

Samples from four replicates of both types of cultures, corresponding to the mid-log phase of growth (condition 2, with an OD_600_ between 10 and 15), were used. We selected this condition because it corresponds to the mature state of the cells that were used in previous biocatalytic reactions that were studied by our group. As mentioned above, notable differences were observed when using a seawater- or freshwater-based medium for this purpose.

**Figure 8:**
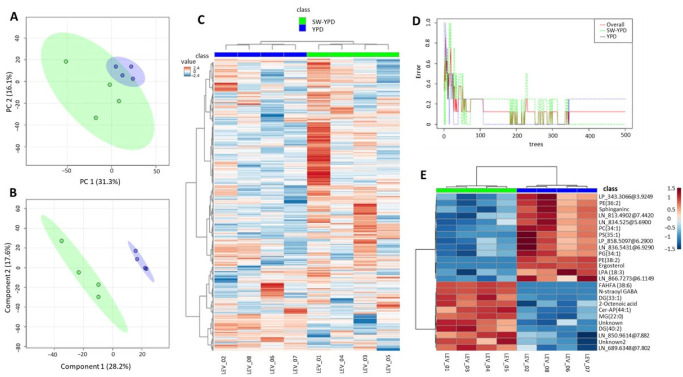
Untargeted lipidomic profiling distinguishes *S. etchellsii* cells grown in SW-YPD from those grown in YPD. (**A**) PCA scores plot of the total lipidome (in green SW-YPD, in blue YPD). (**B**) Partial Least Squares Discriminant Analysis (PLS-DA [32]) with model performance metrics: Accuracy = 0.90, R^2^ = 1.00, and Q^2^ = 0.43. (**C**) Heatmap of Pearson correlation coefficients across samples, showing hierarchical clustering according to growth medium. (**D**) Random Forest classification plot. The Out-Of-Bag (OOB) error is 0.125, and the classification error is 0.0 for SW-YPD and 0.25 for YPD samples. (**E**) Heatmap showing hierarchical clustering of the top 25 most significantly different lipid features (according to *t*-test *p*-values) and their relative intensity values across samples from the two conditions.

Following quality control, filtering, and signal correction, 1963 molecular features (compounds) were used for statistical analysis. Figure 8 shows the results of the multivariate statistics and machine learning model. Supplementary Figure S2 contains heat maps corresponding to the top 100 and 50 compounds of the lipidome for which the greatest differential levels were found between the two tested conditions. According to these data, the global lipidome profiles of the two sets of samples differed greatly. Thus, the samples were clustered separately by experimental group in the PCA. Random Forest could perfectly classify the SW-YPD samples (error of 0.0). Furthermore, univariate statistics (*t*-test with an FDR-adjusted *p*-value of less than 0.05) revealed nine statistically significant differential compounds (Table 10). These compounds are annotated in the Top25 image (Figure 8E), and the differential levels are shown in Supplementary Figure S3. They correspond to various lipid classes and categories, including fatty acyls (FAs), glycerolipids (GLs), such as monoacylglycerols (MGs) and diacylglycerols (DGs), glycerophospholipids (GPs), including serine derivatives (PSs) and ethanolamine derivatives (PEs), sphingolipids (ceramides, Cer), and sterol lipids (ergosterol). GPs and sterol lipids were found at higher levels in cells grown in YPD, while the other compounds were enriched in cells from seawater cultures.

The ergosterol content of these samples was determined using spectroscopic methods. These results were consistent with the lipidomics analysis and revealed a statistically significant difference in ergosterol content between cells grown in YPD and SW-YPD (0.3257 ± 0.0159 and 0.265 ± 0.003 mg/mg dry weight, respectively, *p* = 0.0193). The same analysis was performed on cells undergoing osmotic shock, and again, the samples from cells transferred to seawater had lower ergosterol content (0.4003 ± 0.0664 mg/mg dry weight in YPD and 0.2751 ± 0.031 mg/mg dry weight in SW-YPD, with a *p*-value of 0.0111).

## 3. Discussion

Several reports have described the use of halotolerant yeasts in bioprocesses in seawater media [33,34,35,36]. *S. etchellsii* is an unconventional halotolerant yeast about which little is known at the molecular level, despite recent interest in its biotechnological applications in the pharmaceutical and industrial sectors [5,6,7]. This study conducted comparative proteomic and lipidomic analyses at various growth stages in seawater and freshwater media to elucidate the molecular mechanisms underlying the behavior and potential of *S. etchelsii* in seawater containing approximately 4% (*w*/*v*) salts, primarily sodium chloride. This is the first time that these two omic approaches have been used together with this yeast.

Proteome comparisons were performed throughout the growth curves of *S. etchellsii* in both seawater and freshwater at time points corresponding to the early, middle, and late log phases. One interesting result was the detection of higher levels of several proteins in seawater, including those involved in lipogenesis (e.g., inositol-3-phosphate synthase and phosphatidate cytidylyl transferase), glycerol transport (e.g., Stl1), oxidative stress (e.g., superoxide dismutase and peroxidase), osmotic stress (e.g., glycerol-3-phosphate dehydrogenase and a homologous protein of *S. cerevisiae* Ena1, *M. guilliermondii* A5DHH7_PICGU, and *D. hansenii* ENA1p), and carbohydrate metabolism (e.g., pyruvate carboxylase) (Figure 6 and Supplementary Table S6). In the freshwater medium, however, the expression of proteins involved in biosynthetic processes related to organonitrogen compounds and others associated with the generation of precursor metabolites and energy, carbohydrate metabolism, and lipid metabolism was higher. Certain proteins that respond to stressful conditions are found to be up-regulated in a seawater or freshwater medium. Notably, a recent study by Navarrete et al. investigating the transcriptomic and proteomic differences in *D. hansenii* exposed to 1M NaCl or 1M KCl, also revealed increased levels of Ena1, Stl1, and several glucose and phosphate transporters in the presence of 1M NaCl [19].

Data obtained from applying osmotic stress to *S. etchellsii* cells by transferring a culture grown in a freshwater medium to a seawater-based medium for further incubation (condition 4) also provided useful information. A total of 169 proteins were up-expressed and 77 were down-regulated when considering differential values greater than 1.3 (Table 1). The most relevant changes observed in terms of protein levels were higher expression of enzymes such as glycerol-3-phosphate dehydrogenase, pyruvate carboxylase, and inositol-3-phosphate synthase. Lower levels were detected for proteins involved in biosynthetic processes, such as translation, nitrogen compound metabolism, protein folding, and intracellular protein transport (see Table 8 and Table 9, Figure 5 and Supplementary Table S5). Overall, the proteins up-expressed in seawater under conditions 4 and 1 are most similar. This is the case for the enzymes inositol-3-phosphate synthase and glycerol-3-phosphate dehydrogenase. The latter enzyme is involved in glycerol biosynthesis, a process by which *D. hansenii* produces the preferred compatible solute to counteract hyperosmotic stress. However, it also uses trehalose, arabinitol, glutamic acid, and alanine for this purpose [18].

These data are worth comparing with those of Gori et al., who used a proteomic approach to understand *D. hansenii*’s response to 8% and 12% (*w*/*v*) NaCl [15]. The authors identified six up-regulated and nine down-regulated proteins. Among the upregulated proteins, they found enzymes involved in glycerol metabolism as well as a heat shock protein (Ssa1), a protein involved in the response to oxidative stress (peroxiredoxin Prx1), and enzymes responsible for reactions in the upper part of glycolysis. Regarding the down-regulated proteins, they identified enzymes from the lower part of glycolysis, the Krebs cycle and amino acid biosynthesis. They also found a flavohaemoglobin (Yhb1) and two heat shock proteins (Hsp60 and Ssb2). Similarly, our studies indicated lower expression of some proteins involved in protein folding, such as peptidyl-prolyl cis-trans isomerase, Hsp90, and Pfp1, in a seawater medium. However, proteins involved in the response to oxidative stress, such as monothiol glutaredoxin and glutathione peroxidase, were also found to be down-expressed in seawater. This could indicate a lower degree of oxidative stress in this medium. While some studies have suggested that glutamate plays an important role in *D. hansenii* growth at high NaCl concentrations [37,38], experiments described in this work as well as those of Gori et al. [15], revealed no evidence of increased glutamate dehydrogenase expression under osmotic stress conditions. In fact, we detected higher levels of this enzyme in freshwater than in seawater. It is worth mentioning that we took into account conditions similar to those used in biocatalytic processes previously carried out in our laboratory. Other changes in protein levels under different growth conditions cannot be excluded.

In this proteomic study, we identified an Ena1-like protein in *S. etchellsii* that appears to be conserved in other yeasts, such as *S. cerevisiae*, *D. hansenii*, and *M. guilliermondii* (see Figure 7 and Figure S1). This protein plays an important role in the response to osmotic stress as a P-type Na^+^ transporter. Further similarity analyses with its counterparts in other yeasts will be possible once the *S. etchellsii* protein sequence is available. The increased expression of P-type Na^+^ transporters in yeast grown under high-salt conditions is an expected adaptive response to osmotic stress [39]. These transporters play a crucial role in maintaining intracellular ion homeostasis by actively exporting excess sodium ions, thereby protecting cellular functions and preserving membrane potential. This mechanism is essential for yeast survival in saline environments. From a biotechnological perspective, the presence of this transporter makes these yeast strains highly valuable as biocatalysts for industrial processes conducted under high-salinity or otherwise stressful conditions [39]. Their enhanced salt tolerance enables efficient operation in less controlled containers or reactors with non-sterile or saline media, including seawater-based fermentations, brines, and wastewater. This reduces environmental purification and process costs, as well as contamination risks. Furthermore, these yeast strains can be used in bioremediation or engineered to produce bioactive compounds or biofuels in harsh environments where conventional strains would not survive.

The effect of growth in seawater or freshwater on cell lipid composition was studied under condition 2 (OD_600_ around 15). PCA revealed global differences between the two groups, and the Random Forest algorithm could classify samples in the SW-YPD group. Furthermore, a *t*-test with a *p*-value adjusted by FDR < 0.05 identified nine significant compounds. Six of these compounds (primarily FAs and MGs and DGs) exhibited higher levels in cells cultivated in a seawater medium, while three compounds (PS, PE and ergosterol) were more prevalent in the control group. Spectroscopic determinations confirmed the reduction in ergosterol content in seawater media, which is consistent with previous data for other yeasts, such as *S. cerevisiae* [40], *H. werneckii*, and *D. hansenii* [41]. However, the opposite was found in *Zygosaccharomyces rouxii* [42]. Ergosterol levels in osmoadaptation have been described as related to plasma membrane pump and channel activity, as well as to mechanical properties [43]. Indeed, sterols provide lipid bilayers with additional rigidity and reduced permeability to hydrophilic molecules. Some authors have described morphological changes, such as deformations and invaginations, in yeast cells exposed to salt stress. It has been postulated that rapid protein internalization may occur under these conditions [44]. Using model lipid membranes, Sokolov et al. showed that decreasing sterol content increases water permeability, prevents osmotic pressure-induced plasma membrane rupture, and allows for a faster recovery from the transient loss of barrier function [43]. The results obtained in *S. cerevisiae*, *D. hansenii*, *H. werneckii*, and now *S. etchellsii*, show that osmoadaptation in these yeasts is associated with increased plasma membrane fluidity.

**Figure 9:**
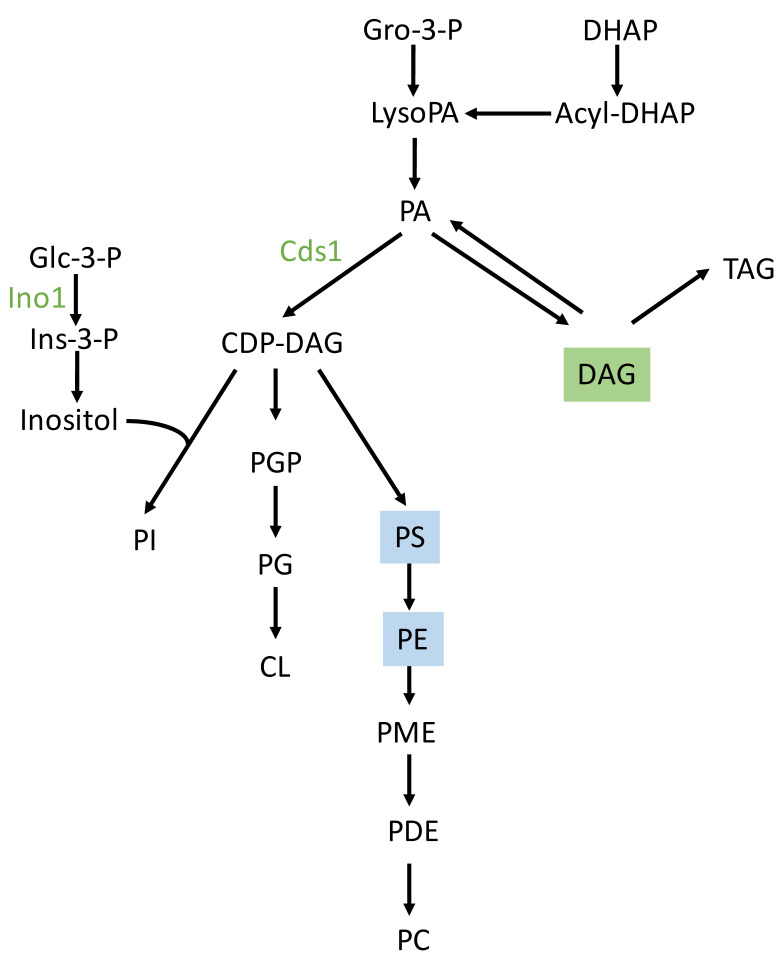
Schematic of the CDP-DAG pathway of phospholipid synthesis in *S. cerevisiae*. Adapted from [45]. Abbreviations: CDP-DAG, CDP-diacylglycerol; CL, cardiolipin; Gro, glycerol; DHAP, dihydroxyacetone phosphate; Glc, glucose; Ins, inositol; PA, phosphatidate; PC, phosphatidylcholine; PE, phosphatidylethanolamine; PG, phosphatidylglycerol; PGP, phosphatidylglycerophosphate; PDE, phosphatidyldimethylethanolamine; PI, phosphatidylinositol; PME, phosphatidylmonomethylethanolamine; PS, phosphatidylserine; TAG, triacylglycerol. Cds1 is the phosphatidate cytidylyltransferase (CDP-diacylglycerol synthetase). The proteins in green color are overexpressed in seawater under some of the conditions described in this work. Lipids shaded in green and blue correspond to those with higher levels in seawater and freshwater, respectively.

Although proteomic analyses under seawater conditions revealed higher levels of phosphatidate cytidylyltransferase (Cds1) and inositol 3-phosphate synthase, non-targeted lipidomics analysis found no difference in phosphatidylinositol (PI) content between the two environments. However, higher levels of PS and PE were found in freshwater. Cds1 catalyzes the conversion of phosphatidic acid (PA) to cytidine diphosphate-diacylglycerol (CDP-DAG), which is an essential intermediate in the synthesis of phosphatidylglycerol (PG), cardiolipin (CL) and PI. According to the CDP-DAG pathway for phospholipid biosynthesis in yeast (Figure 9) and the Kennedy pathway [45] for the biosynthesis of phospholipids other than PI, the proteomics and lipidomics data could suggest metabolic shifts towards PI biosynthesis in a seawater environment, and towards PE and PS biosynthesis in a freshwater environment. However, further analyses are needed to confirm this possibility. An increase in PI synthesis in a seawater environment could boost the production of inositol polyphosphates (InsPs) and inositol pyrophosphates (PP-InsPs). These are key signaling molecules involved in the response to osmotic stress, nutrient sensing, and energy homeostasis [46].

In conclusion, the results of this study provide insights into the physiological and biotechnological characteristics of *S. etchellsii* and its ability to adapt to high-salinity environments. These results are consistent with previously reported osmoadaptive strategies in related yeast studies. These strategies include systematic changes in membrane lipid composition and protein expression related to glycerol metabolism, ion transport, oxidative and osmotic stress, and central carbon metabolism.

Although *S. etchellsii* is an osmotolerant yeast, Figure 1 shows that it grows better in freshwater than in seawater until the stationary phase. The higher overall expression of proteins involved in biosynthetic processes in freshwater (Figure 2, Figure 3 and Figure 4) is consistent with this observation.

Some of the comparisons considered in this work revealed that several proteins appear to be overexpressed, which could explain the observed differences in reactions catalyzed by *S. etchellsii* in seawater or freshwater. For example, the overexpression of several alcohol/aldehyde dehydrogenases in seawater could explain the improved outcomes of reduction reactions observed in previous studies [6,7]. However, the results of this study suggest that the increased activity observed in seawater may be due to factors other than levels of these catalytic proteins. For example, the overexpression of osmotic stress response proteins could account for the cells’ higher resistance to increased organic substrate loads and greater capacity for reuse. Decreased ergosterol content in cells grown in seawater results in increased fluidity and decreased barrier function [43]. This could favor biocatalytic processes by facilitating substrate diffusion. Changes in other aspects of the cell membrane, such as phospholipid composition, may explain why cells grown in seawater are more thermotolerant and resistant to organic compounds.

Further experiments are needed to better understand the relationship between the proteomic and membrane changes identified in these multiomic studies and the observed phenotypes and behavior of *S. etchelsii*. In the future, it would be especially interesting to determine the role of the ion transporters and delve deeper into the stress response pathways. Additionally, a more in-depth study of changes in lipid species under diverse salt concentrations should be undertaken.

## 4. Materials and Methods

### 4.1. Strains and Growth Conditions

The yeast strain used in this study, *S. etchellsii* (CECT 11406), was obtained from the “Colección Española de Cultivos Tipo, Servei Central de Suport a la Investigació”, Universitat de València, Paterna (Spain). The strain was isolated from fermenting cucumbers.

This strain was grown in a solution containing 1% yeast extract, 2% bactopeptone, and 2% glucose. The solution was prepared using either freshwater (YPD) or seawater (SW-YPD). For most experiments, the cells were incubated overnight in YPD or SW-YPD at 30 °C in an orbital shaker set to 200 rpm. The cultures were then diluted in the same medium to a final OD_600_ of 0.3, after which growth continued. Samples were taken at different time points to track growth and perform proteomic and lipidomic analyses.

To determine the effect of switching from a freshwater to a seawater medium, the extracts were obtained from cultures prepared as follows: First, the cells were grown in YPD to an OD_600_ of 7. Then, equal amounts of cells were collected and transferred to YPD and SW-YPD. The cultures were grown for three hours until the OD_600_ reached approximately 15 and 16 for the seawater and freshwater cultures, respectively. The cells were then collected, and the extracts were prepared.

The seawater used in the experiments was collected from El Perelló Beach in the Mediterranean Sea in València, Spain. It was sterilized by autoclaving under standard conditions. The salinity of the water in this area was approximately 4% (*w*/*v*), as determined by weighing the solid residue after lyophilization. The sodium content was analyzed using Flame Emission Spectroscopy (589.0 nm; Thermo Fisher Scientific, Waltham, MA, USA, iCE 3000 Series) and was found to be 12.46 ± 0.17 g/L. The pH ranged from 7.5 to 8.5 depending on the batch.

### 4.2. Proteome Analyses

Aliquots of *S. etchellsii* cultures in seawater and freshwater were taken at time points corresponding to OD_600_ values of 2–3, 15–20, and 45–50. The cultures were then centrifuged and cells lysed as previously described [9]. As explained above, other cultures were grown in YPD up to an OD_600_ value of 7 and then subjected to an osmotic shock before extract preparation. Experiments were carried out in triplicate.

Proteomic analyses were performed on these samples at the Proteomics Facility of the SCSIE (Universitat de València) using the Sequential Window Acquisition of all Theoretical Mass Spectra methodology (SWATH-MS) [47]. The procedure described in Andreu and del Olmo [9] was followed for the preparation and analysis of the joint spectral library and the individual samples in each experiment. To analyze the functional categories involved in expression changes, an interaction and enrichment analysis was carried out using the Search Tool for the Retrieval of Interacting Genes/Proteins (STRING) [48]. Proteins with an unused score greater than 1.3 were identified with at least 95% confidence.

The mass spectrometry proteomics data have been deposited in the ProteomeXchange Consortium via the PRIDE partner repository [49]. The dataset identifiers are PXD070465, PXD070554 and PXD070596.

The proteomes were functionally analyzed using data from the UniProt Knowledgebase, which contains annotated UniProtKB/Swiss-Prot entries [22]. Functional Gene Ontology (GO) terms were assigned to each identified protein based on yeast genome data. The “biological process” hierarchy was primarily used to analyze differentially expressed proteins.

Protein sequence alignments were performed using the European Molecular Biology Open Software Suite (EMBOSS 6.5.7) Matcher facility [28] and the Clustal Omega (CLUSTAL O) (1.2.4) multiple sequence alignment of the National Center of Biotechnology Information (NCBI) protein-protein BLAST service [29].

### 4.3. Lipid Analyses

Non-targeted lipidomic analyses were performed as described by Obis et al. [50], with the following modifications. Total lipids were isolated from a quantity of cells equivalent to five OD_600_ units. The cells were grown in a seawater or freshwater medium until reaching an OD_600_ of 10–15. Four replicates were made for each condition. To preserve the cells’ physiological metabolism, quenching was performed by incubation in cold methanol at −45 °C. The cells were then collected by centrifugation and stored at −80 °C until lipid extraction. The cell pellets were resuspended in 200 µL of cold PBS and sonicated for 10 s using a Soniprep 150 Ultrasonic Desintegrator (MSE, London, UK). An aliquot of 10 µL of the cell homogenate was used for lipid extraction by adding 25 µL of ice-cold MiliQ water:methanol (1:4) and 250 µL of methyl tert-butyl ether (MTBE) containing internal lipid standards (see Supplementary Table S1). The samples were sonicated again in a water bath (ATU Ultrasonidos, València, Spain) at a frequency of 40 kHz and power of 100 W for 30 min at 10 °C. Then, 25 μL of MiliQ water was added to the mixture, and the organic phase was centrifuged at 1400 g and 10 °C for 10 min. The lipid extracts in the upper phase were then subjected to mass spectrometry. A pool of all lipid extracts was prepared and used as a quality control sample. Internal, isotopically labeled lipid standards for each class were used for signal normalization. Stock solutions of the internal standards were prepared by dissolving the lipid standards in MTBE at a concentration of 1 mg/mL. Working solutions were then diluted to 2.5 μg/mL in MTBE.

Lipid extracts were analyzed using liquid chromatography–mass spectrometry with a UHPLC 1290 series coupled to an ESI-Q-TOF MS/MS 6545 (Agilent Technologies, Barcelona, Spain). For each sample, 10 μL of the lipid extract was injected into a Waters Acquity HSS T3 column (1.8 μm particle size, 100 × 2.1 mm id, Waters, Milford, MA, USA), which was heated at 55 °C. The flow rate was set at 400 μL/min. Solvent A contained 10 mM ammonium acetate in a 40:60 (*v*/*v*) acetonitrile–water mixture, and solvent B contained 10 mM ammonium acetate in a 10:90 (*v*/*v*) acetonitrile–isopropanol mixture. The gradient started at 40% solvent B and reached 100% B within 10 min, holding for two minutes. Finally, the system switched back to 40% solvent B, equilibrating for 3 min. Duplicate runs of the samples were performed to collect positive and negative electrospray-ionized lipid species in TOF mode. The TOF was operated in full-scan mode at 100 to 1700 *m/z* and used N_2_ as the nebulizer gas at 5 L/min and 350 °C. The capillary voltage was set to 3500 V, and one scan per second was acquired. A double spray with reference solution was used for continuous infusion and in-run calibration of the mass spectrometer [50]. For preprocessing and annotation, features with at least two ions present in 70% of an experimental group’s samples were selected. The retention time and mass windows were set to 0.1% ± 0.25 min and 30.0 ppm ± 2.0 mDa, respectively, using MassHunter Profinder 10.0 software (Agilent Technologies, Barcelona, Spain). We used MassHunter Mass Profiler Professional 15.1 software (Agilent Technologies, Barcelona, Spain) and MetabolAnalyst 6.0 software to perform a non-targeted lipidomic analysis of the obtained data [51,52]. For annotation, relevant features, defined by exact mass, retention time and MS/MS spectra, were searched against the Human Metabolome Database (HMDB) [53], LIPID MAPS databases (accuracy <20 ppm) [54] and LipidMatch [55]. The identities obtained were compared to the authentic standards’ retention times.

Untargeted lipidomic analysis in yeast was assessed using univariate and multivariate statistical approaches to identify changes in lipid profiles under different conditions. A *t*-test was performed on each lipid species for univariate analysis to detect statistically significant changes between groups. *p*-values were adjusted for multiple testing using the Benjamini–Hochberg method to control the false discovery rate [56].

The determination of ergosterol followed the methods described by Zuzuarregui et al. [57].

## Figures and Tables

**Table 1:** Overall results of proteomic comparison at different time points of *S. etchellsii* growth curve in SW-YPD and YPD.

Condition	Spectral Library ^1^	Differentially Expressed (>1.3) ^2^
**1**: SW-YPD vs. YPD at OD_600_ around 2–3	1762	155 up-expressed 153 down-expressed
**2**: SW-YPD vs. YPD at OD_600_ around 15–20	1744	170 up-expressed 244 down-expressed
**3**: SW-YPD vs. YPD at OD_600_ around 45–50	2330	4 up-expressed 11 down-expressed
**4**: SW-YPD vs. YPD after initial growth in YPD	2166	169 up-expressed 77 down-expressed

^1^ Number of proteins identified. ^2^ Ratio between the values found in SW-YPD and YPD.

**Table 2:** Different proteins up-expressed at least 2-fold higher in seawater than in freshwater medium after incubation in YPD or SW-YPD up to OD_600_ = 2–3.

Protein Organism Recommended Name	Biological Process	Similar Proteins ^1^	Fold Change (Seawater/Freshwater)
W1QHM6_OGAPD *Ogatea parapolymorpha* Inositol-3-phosphate synthase	Lipid metabolic process: inositol/phospholipid biosynthetic process	Inositol-3-phosphate synthase (*Hansenula polymorpha*, 90%)	4.38
Q6BQS9_DEBHA *D. hansenii* DEHA2E02574p	Generation of precursor metabolites and energy		3.41
G3AW91_CANTC *Candida tenuis* Alcohol dehydrogenase			2.79
A5DHH7_PICGU *Meyerozyma guilliermondii* P-type Na(+) transporter	Transmembrane transport: potassium and sodium ion transport	P-type Na(+) transporter (*Meyerozyma* sp. JA9, 90%)	2.47
A5DBS5_PICGU *M. guilliermondii* Aspartate-semialdehyde dehydrogenase	Lipid, sulfur compound and amino acid metabolic process: amino acid (Lys, Met, Thr) biosynthesis	Aspartate-semialdehyde dehydrogenase (*Meyerozyma* sp. JA9, 90%)	2.35
A0A512U616_9ASCO *Metschnikowia* sp. JCM33374 J domain-containing protein	Protein refolding: chaperone cofactor-dependent protein refolding; response to heat		2.16
Q6BN36_DEBHA *D. hansenii* DEHA2F00572p	Transmembrane transport: glycerol transmembrane transport		2.09

^1^ Only those proteins that have at least 90% identity are shown.

**Table 3:** Different proteins up-expressed at least 2-fold higher in freshwater than in seawater medium after incubation in YPD or SW-YPD up to OD_600_ = 2–3.

Protein Organism Recommended Name	Biological Process	Similar Proteins ^1^	Fold Change (Seawater/Freshwater)
H8X2Z1_CANO9 *Candida orthopsilosis* Thiamine thiazole synthase	>Vitamin metabolic process: thiamine biosynthesis	>Thiamine thiazole synthase (*Candida parapsilosis*, *Candida metapsilosis*, 90%)	>0.05
Q6BMZ8_DEBHA *D. hansenii* Pyridoxal 5′-synthase (glutamine hydrolyzing)	Amino acid and vitamin metabolic process: pyridoxine biosynthesis	Pyridoxal 5′-synthase (glutamine hydrolyzing) (*Debaryomyces fabryi*, 90%)	0.35
C5DBT9_LACTC *Lachancea thermotolerans* KLTH0A05302p	Nucleobase-containing small molecule metabolic process: nucleotide metabolic process	LAQU0S09e01926g1_1 (*Lachancea quebecensis*, 90%)	0.36
H8X0F9_CANO9 *C. orthopsilosis* Cdc12 septin		Septin-type G domain-containing protein (*C. parapsilosis*), Septin family protein (*C. parapsilosis*), CDC12 (*C. metapsilosis*, *Candida theae*), 90%	0.40
G8YRV7_PICSO *Pichia sorbitophila* Guanylate kinase	Nucleobase-containing small molecule metabolic process; carbohydrate derivative metabolic process	Guanylate kinase (*P. sorbitophila*, 90%)	0.44
C5MJ06_CANTT *Candida tropicalis* Adenosylhomocysteinase	Amino acid, sulfur compound, carbohydrate, nucleobase-containing small molecule and lipid metabolic process: S-adenosylmethionine cycle, phosphatidylcholine biosynthetic process	Adenosylhomocysteinase (several yeasts, 90%)	0.46
A0A1E5S0H6_HANUV *Hanseniaspora uvarum* Fructose-bisphosphate aldolase	Carbohydrate metabolic process; generation of precursor metabolites and energy; nucleobase- containing small molecule metabolic process: gluconeogenesis; glycolytic process	Fructose-bisphosphate aldolase (*Hanseniaspora opuntiae*, *Hanseniaspora valbyensis NRRL Y-1626,* *Hanseniaspora guilliermondii,* 90%)	0.50

^1^ Only those proteins that have at least 90% identity are shown.

**Table 4:** Different proteins up-expressed at least 2-fold higher in seawater than in freshwater medium after incubation in YPD or SW-YPD up to OD_600_ = 15–20.

Protein Organism Recommended Name	Biological Process	Similar Proteins ^1^	Fold Change (Seawater/Freshwater)
H8WZA6_CANO9 *C. orthopsilosis* Superoxide dismutase [Cu-Zn]		Superoxide dismutase [Cu-Zn] (in several *Candida* species, 90%)	3.14
M3HGM4_CANMX *Candida maltosa* Phosphatidate cytidylyltransferase	Lipid metabolic process: CDP-diacylglycerol biosynthesis		2.78
Q9C1R0_DEBHN *D. hansenii* P-type Na(+) transporter	Transmembrane transport: sodium and potassium transport	P-type Na(+) transporter (*D. fabryi, D. hansenii*), 90%	2.08

^1^ Only those proteins that have at least 90% identity are shown.

**Table 5:** Different proteins up-expressed at least 2-fold higher in freshwater than in seawater medium after incubation in YPD or SW-YPD up to OD_600_ = 15–20.

Protein Organism Recommended Name	Biological Process	Similar Proteins ^1^	Fold Change (Seawater/Freshwater)
A0A4P6XSF2_9ASCO *Metschnikowia aff. pulcherrima* Thiamine thiazole synthase	Vitamin and sulfur compound metabolic process: thiamine/thiazole biosynthetic process	Thiamine thiazole synthase (*M. pulcherrima*, 100%; *Metschnikowia bicuspidata*, *Metschnikowia* sp. JCM33374, 90%)	0.016
Q6CSN8_KLULA *Kluyveromyces lactis* 4-amino-5-hydroxymethyl-2-methylpyrimidine phosphate synthase	Vitamin and sulfur compound metabolic process: thiamine biosynthesis		0.077
G8YLA6_PICSO *P. sorbitophila* Polyadenylate-binding protein	Translation regulation/Transport	Polyadenylate-binding protein (*P. sorbitophila*, 90%)	0.151
C5DNS8_LACTC *L. thermotolerans* L-lactate dehydrogenase	Lactate/pyruvate metabolic process	L-lactate dehydrogenase (*L. quebecensis*, 90%)	0.186
C4YMM9_CANAW *C. albicans* Aconitate hydratase, mitochondrial	Generation of precursor metabolites and energy: tricarboxylic acid cycle	Aconitate hydratase, mitochondrial (*Teunomyces kruisii* and several *Candida* species, 90%)	0.353
Q6BMZ9_DEBHA *D. hansenii* Glutaminase	Amino acid and vitamin metabolic process: glutamine metabolic process, pyridoxine metabolic process.		0.353
R9XDR8_ASHAC *Ashbya aceri* AaceriAEL253Wp	Mitochondrion organization: mitochondrial genome maintenance; transmembrane transport: pyrimidine nucleotide import into mitochondrion	AEL253Wp (*Eremothecium gossypii*, 90%)	0.362
B9WM32_CANDC *Candida dubliniensis* Adenylosuccinate lyase	Nucleobase-containing small molecule metabolic process: “de novo” AMP and IMP biosynthesis	Adenylosuccinate lyase (*Saccharomyces elongisporus* and several *Spathaspora* and *Candida* species, 90%)	0.414
A0A0H5C3C5_CYBJN *Cyberlindnera jadinii* Glutathione peroxidase	Cellular response to oxidative stress		0.452
Q6BTD9_DEBHA *D. hansenii* Ferric-chelate reductase (NADPH)	Copper ion import; Iron ion transport		0.453
G8YRV7_PICSO *P. sorbitophila* Guanylate kinase	Nucleobase-containing small molecule metabolic process; carbohydrate derivative metabolic process		0.473
A5DBA7_PICGU *M. guilliermondii* Serine/threonine-protein phosphatase	Mitotic cell cycle; chromatin organization; regulation of DNA-template transcription; chromosome segregation; reproductive process; meiotic nuclear division	Serine/threonine-protein phosphatase (*C. tenuis*, *M. pulcherrima*, 90%)	0.483
G3B8S6_CANTC *C. tenuis* 60S ribosomal protein L35	Ribosome biogenesis; translation		0.489
Q5A8A6_CANAL *C. albicans* Carbamoyl-phosphate synthase (ammonia)	Amino acid metabolic process: L-arginine biosynthesis; nucleobase-containing small molecule metabolic process: pyrimidine nucleotide biosynthesis	Carbamoyl-phosphate synthase (ammonia) (*C. albicans* strain WO-1, 100%; *C. albicans* P78048, 90%)	0.491

^1^ Only those proteins that have at least 90% identity are shown.

**Table 6:** Different proteins up-expressed at least 2-fold higher in seawater than in freshwater medium after incubation in YPD or SW-YPD up to OD_600_ = 45–50.

Protein Organism Recommended Name	Biological Process	Similar Proteins ^1^	Fold Change (Seawater/Freshwater)
A5DHH7_PICGU *M. guilliermondii* P-type Na(+) transporter	Transmembrane transport: sodium and potassium transport	P-type Na(+) transporter (*Meyerozyma* sp. JA9, 90%)	4.55
A5DIA7_PICGU *M. guilliermondii* Aldehyde dehydrogenase domain-containing protein	Acetate biosynthetic process		2.08

^1^ Only those proteins that have at least 90% identity are shown.

**Table 7:** Different proteins up-expressed at least 2-fold higher in freshwater than in seawater medium after incubation in YPD or SW-YPD up to OD_600_ = 45–50.

Protein Organism Recommended Name	Biological Process	Similar Proteins ^1^	Fold Change (Seawater/Freshwater)
C4Y6B1_CLAL4 *Clavispora lusitaniae* Phosphoglycerate kinase	Generation of precursor metabolites and energy; nucleobase containing small molecules metabolic process; carbohydrate metabolic process: glycolysis and gluconeogenesis	Phosphoglycerate kinase (*C. lusitaniae*, 100%; several other yeasts, 90%)	0.03
G3BBB4_CANTC *C. tenuis* 40S ribosomal protein S4	Translation	40S ribosomal protein S4 (several yeasts, 90%)	0.09
F2QXG9_KOMPC *Komagataella phaffii* Phorphopyruvate hydratase	Generation of precursor metabolites and energy; nucleobase containing small molecules metabolic process; carbohydrate metabolic process: glycolysis	Phorphopyruvate hydratase (*K. phaffii*, 100%; *Komagataella pastoris*, 90%)	0.15
A0A512U9M4_9ASCO *Metschnikowia* sp. JCM 33374 Glyceraldehyde-3-phosphate dehydrogenase	Generation of precursor metabolites and energy; nucleobase containing small molecules metabolic process; carbohydrate metabolic process: glycolysis	Glyceraldehyde-3-phosphate dehydrogenase (several yeasts, 90%)	0.17
A5DKU4_PICGU *M. guilliermondii* Glutamate dehydrogenase	Amino acid metabolic process: glutamate biosynthesis	Glutamate dehydrogenase (*Meyerozyma* sp. JA9, 90%)	0.35
A5DP35_PICGU *M. guilliermondii* Piridoxamine kinase/Phosphomethylpyrimidine kinase domain-containing protein	Vitamin and sulfur compound metabolic process: thiamine biosynthetic process		0.42

^1^ Only those proteins that have at least 90% identity are shown.

**Table 8:** Different proteins up-expressed at least 2-fold higher in seawater than in freshwater medium 3 h after a sudden transfer of YPD growing cells (OD_600_ = 7) from YPD to SW-YPD or YPD.

Protein Organism Recommended Name	Biological Process	Similar Proteins ^1^	Fold Change (Seawater/Freshwater)
A0A1Z8JNR8_PICKU *Pichia kudriavzevii* Carbamoyl-phosphate synthase (ammonia)	Small molecule metabolic process; signaling: G protein coupled receptor signaling pathway		3.867
G8Y2N4_PICSO *P. sorbitophila* DNA mismatch repair protein	DNA repair: mismatch repair	DNA mismatch repair protein (*P. sorbitophila*, 90%)	2.753
C4Y4H0_CLAL4 *C. lusitaniae* Pyruvate carboxylase	Carbohydrate metabolic process: gluconeogenesis, pyruvate metabolism	Pyruvate carboxylase (*C. lusitaniae*, 100%; several yeasts, 90%)	2.403
M3JZL6_CANMX *C. maltosa* Alpha-glucosidase	Carbohydrate metabolic process: maltose, sucrose catabolic process		2.402
B5TYI1_SCHSH *Scheffersomyces shehatae* Xylitol dehydrogenase	Carbohydrate metabolism: sorbitol catabolic process		2.326
N1P775_YEASC *Saccharomyces cerevisiae* Serine/threonine protein phosphatase	Chromatin reorganization; regulation of DNA-templated transcription	Serine/threonine protein phosphatase (several *Saccharomyces* species, 100 or 90%)	2.251
G3AWW0_CANTC *C. tenuis* Inositol-3-phosphate synthase	Lipid (phospholipid) metabolic process: inositol biosynthesis		2.208
G3BE53_CANTC *C. tenuis* Multicatalytic endopeptidase			2.170
W0TYS3_DEBHA, *D. hansenii* Glycerol-3-phosphate dehydrogenase [NAD(+)]	Carbohydrate metabolic process: glycerol-3-phosphate catabolism	Glycerol-3-phosphate dehydrogenase [NAD(+)] (*D. hansenii*, 100%; several *Debaryomyces* species, 90%)	2.053

^1^ Only those proteins that have at least 90% identity are shown.

**Table 9:** Different proteins up-expressed at least 2-fold higher in freshwater than in seawater medium 3 h after a sudden transfer of YPD growing cells (OD_600_ = 7) from YPD to SW-YPD or YPD.

Protein Organism Recommended Name	Biological Process	Similar Proteins ^1^	Fold Change (Seawater/Freshwater)
C4YBA5_CLAL4 *C. lusitaniae* Malic enzyme	Pyruvate, malate and amino acid metabolism	Malate dehydrogenase (*C. lusitaniae*, 90%)	0.072
A7TI55_VANPO *Kluyveromyces polysporus* Dipeptidyl peptidase 3	Proteolysis		0.158
A0A1E3P6Z8_WICAA *Hansenula anomala* Peptidyl-prolyl *cis*-*trans* isomerase	Regulation of DNA-templated transcription; protein folding; reproductive process; cell differentiation; anatomical structure development; meiotic nuclear division	Peptidyl-prolyl *cis*-*trans* isomerase (*Wickerhamomyces mucosus*, 90%)	0.255
A0A1E5RXI8_HANUV *Kloeckera apiculata* Guanylate kinase	Nucleobase containing small molecule metabolic process; carbohydrate derivative metabolic process	Guanylate kinase (*H. guilliermondii*, *H. opuntiae*, 90%)	0.337
A3LNI0_PICST *Pichia stipitis* ATP synthase subunit 5, mitochondrial	Nucleobase containing small molecule metabolic process; carbohydrate derivative metabolic process; transmembrane transport: hydrogen ion transport; ATP synthesis		0.338
A0A1V2L4N1_CYBFA *Hansenula fabianii* Profilin	DNA-templated transcription: transcription elongation by RNA polymerase II; sequestering of actin monomers		0.346
Q6BHS8_DEBHA *D. hansenii* DEHA2G16082p	Translation	Ubiquitin-like domain-containing protein (several yeasts, 90%)	0.346
A0A0H5C3C5_CYBJN *C. jadinii* Glutathione peroxidase	Cellular response to oxidative stress		0.346
Q6BQV0_DEBHA *D. hansenii* ADP-ribosylation factor	Protein transport: vesicle mediated transport	ADP-ribosylation factor (*D. fabryi*, 100%; several yeasts 90%)	0.369
A5DE91_PICGU *M. guilliermondii* Inorganic phosphate transporter Pho88	Protein targeting to ER	Inorganic phosphate transport protein Pho88 (*Meyerozyma* sp. JA9, 90%)	0.369
H2AVL6_KAZAF *Kluyveromyces africanus* Adenylate kinase	DNA replication (initiation): pre-replicative complex assembly; nucleobase containing small molecule metabolic process: ADP biosynthetic process, AMP and ATP metabolic process		0.370
A5DNA3_PICGU *M. guilliermondii* Synaptobrevin homolog Ykt6	Intracellular protein transport; autophagy; vesicle-mediated transport; membrane organization	Synaptobrevin homolog Ykt6 (several yeasts, 90%)	0.376
M3K3L9_CANMX *C. maltosa* Glycosyl hydrolase, putative	Carbohydrate metabolic process; cell wall organization or biogenesis		0.377
Q59PW1_CANAL *C. albicans* Monothiol glutaredoxin	Cellular response to oxidative stress; protein maturation by [2Fe-2S] or [2Fe-4S] cluster transfer; response to osmotic stress	Monothiol glutaredoxin-5, mitochondrial (*C. albicans* P78048, 100%); Grx4 family monothiol glutaredoxin (*C. albicans*, 100%); glutaredoxin domain-containing protein (*C. albicans* strain WO-1, 90%)	0.416
Q6BL76_DEBHA *D. hansenii* DEHA2F15774p	Protein folding: protein stabilization, response to unfolded protein; transmembrane transport and mitochondrion organization: protein import into mitochondrial matrix		0.419
Q6BND9_DEBHA *D. hansenii* Cyclin-dependent kinases regulatory subunit	Cell cycle: cell division	Cyclin-dependent kinases regulatory subunit (several yeasts, 90%)	0.433
A0A1B2J571_PICPA *Pichia pastoris* BA75_00168T0	Protein folding	Heat shock protein regulator (*P. pastoris*, 90%); co-chaperone that binds to Hsp82p and activates its ATPase activity (*P. pastoris*, 90%)	0.460
A0A512UCZ2_9ASCO *Metschnikowia* sp. JCM 33374 Cyclin-dependent kinases regulatory subunit	Cell cycle: cell division	Cyclin-dependent kinases regulatory subunit (*M. bicuspidata* var. *bicuspidata* NRRL YB-4993, 90%)	0.462
Q6CSI7_KLULA *K. lactis* D-lactate dehydrogenase	Methylglyoxal catabolic process to D-lactate via S-lactolyl-glutathione	D-lactate dehydrogenase (*K. lactis*, 90%)	0471
G8B644_CANPC+ *C. parapsilosis* HTH cro/C1-type domain-containing protein	Regulation of DNA-templated transcription: positive regulation of transcription by RNA polymerase II; maintenance of translational fidelity: rescue of stalled ribosome	HTH cro/C1-type domain-containing protein (*C. parapsilosis*, 100%); multiprotein bridging factor 1 (several *Candida* species), 90%	0.498

^1^ Only those proteins that have at least 90% identity are shown.

**Table 10:** Lipid compounds with significant ^1^ differential levels between cells grown in seawater and freshwater media ^2^ up to OD_600_ of 10–15.

Entry Feature ^3^	Adjusted *p*-Value	Putative Compound	Category, Main Class	Subclass
1 LN_582.4609@2.482	5.43 × 10^−7^	FAHFA(38:6)	Fatty Acyls, Fatty esters	Fatty acid estolides
2 LP_391.3036@3.1259997	5.43 × 10^−7^	N-stearoyl GABA	Fatty Acyls, Fatty amides	N-acyl amines
3 LP_639.5278@5.3980002	0.0037598	DG(33:1)	Glycerolipids, Diradylglycerols	Diacylglycerols
4 LP_676.5853@7.3870006	0.02418	DG(40:2)	Glycerolipids, Diradylglycerols	Diacylglycerols
5 LN_769.699@7.777	0.028707	Cer-AP(t44:1)	Sphingolipids, Ceramides	N-acyl-4-hydroxy-sphinganines (phytoceramides)
6 LP_446.3965@5.822999	0.020938	MG(22:0)	Glycerolipids, Monoradylglycerols	Monoacylglycerols
7 LN_775.5353@7.0459995	0.0351	PS(35:1)	Glycerophospholipids, Glycerophosphoserines	Diacylglycerophosphoserines
8 LN_771.5764@7.995	2.39 × 10^−5^	PE(38:2)	Glycerophospholipids, Glycerophosphoethanolamines	Diacylglycerophosphoethanolamines
9 LP_378.327@7.427001	5.43 × 10^−7^	Ergosterol	Sterol Lipids, Sterols	Ergosterols and C24-methyl derivatives

^1^ According to *t*-test with *p*-value adjusted by FDR < 0.05. ^2^ Green and blue shading corresponds to the presence of compounds at higher levels in seawater and freshwater, respectively. ^3^ Polarity mode (LN, negative polarity mode; LP, Positive polarity mode)_Exact mass@Retention time (min).

## Data Availability

Data supporting reported results have been deposited to the ProteomeXchange Consortium via the PRIDE partner repository with the dataset identifiers PXD070465, PXD070554 and PXD070596.

## Supplementary Materials

The supporting information can be downloaded at: https://www.mdpi.com/article/10.3390/ijms27010183/s1.

## Abbreviations

The following abbreviations are used in this manuscript:

BLAST	Basic Local Alignment Search Tool
CDP-DAG	Cytidine Diphosphate–Diacylglicerol
Cer	Ceramides
Cds1	Phosphatidate Cytidylyltransferase
CL	Cardiolipin
DGs	Diacylglicerols
DHAP	Dihydroxyacetone Phosphate
EMBOSS	European Molecular Biology Open Software Suite
ESI-Q-TOF MS/MS	Electrospray Ionization Quadrupole Time-Of-Flight Mass Spectrometry/Mass Spectrometry
FAs	Fatty Acids
FDR	False Discovery Rate
GLs	Glycerolipids
Glc	Glucose
GPs	Glycerophospholipids
Gro	Glycerol
HMDB	Human Metabolome Database
Ins	Inositol
MGs	Monoacylglycerols
MTBE	Methyl Tert-Butyl Ether
NCBI	National Center of Biotechnology Information
OD_600_	Optical Density at a wavelength of 600 nm
PA	Phosphatidate
PBS	Phosphate-Buffered Saline
PC	Phosphatidylcholine
PCA	Principal Component Analysis
PDE	Phosphatidyl Dimethyl Ethanolamine
PEs	Glycerophospholipids (Etahnolamine derivatives)
PG	Phosphatidylglycerol
PGP	Phosphatidylglycerophosphate
PI	Phosphatidylinositol
PLS-DA	Partial Least Squares Discriminant Analysis
PME	Phosphatidylmonomethylethanolamine
PS	Phosphatidylserine
STRING	Search Tool for the Retrieval of Interacting Genes/Proteins
SW	Seawater
TAG	Triacylglycerol
TOF	Time-Of-Flight
UHPLC	Ultra-High-Performance Liquid Chromatography
UniProt tools	Universal Protein Resources
YPD	Yeast Extract Peptone Dextrose
