# Supplementary material for: Unravelling the Molecular Responses of the Yeast Schwanniomyces etchellsii to Hyperosmotic Stress in Seawater Medium Using Omic Approaches
Source: Int J Mol Sci. 2025 Dec 23;27(1):183. doi: 10.3390/ijms27010183 (PMC12785990; doi:10.3390/ijms27010183)
Supplement: Supplementary file 1 [file ijms-27-00183-s001.zip › ijms-4023517-supplementary.pdf]

Supplementary Material to

Unravelling the molecular responses of the yeast *Schwanniomyces etchellsii* to hyperosmotic stress in seawater medium using omic approaches

Index

Supplementary Table S1. Class representative internal standards

Supplementary Table S2. Proteins differentially expressed at OD_{600} 2-3

Supplementary Table S3. Differentially expressed proteins at OD_{600} 15-20

Supplementary Table S4. Proteins with differential expression levels at OD_{600} 45-50

Supplementary Table S5. Differentially overexpressed proteins after osmotic shock

Supplementary Table S6. List of proteins with differential expression levels at least 1.5-fold higher under more than one of the conditions considered in this work

Supplementary Table S7. Homologous proteins in *D. hansenii*, *M. guilliermondii* and *S. cerevisiae* to proteins differentially up-expressed in seawater in the experiments described in this work.

Supplementary Figure S1. Sequence alignment of Ena1 homologs in *S. cerevisiae* and in several non-*Saccharomyces* yeasts with the sequence obtained from the proteomic analysis carried out in *S. etchellsii*.

Supplementary Figure S2. Heatmaps showing the relative abundance of lipid species with the most significant differences between *S. etchellsii* cells grown in SW-YPD and YPD.

Supplementary Figure S3. Differential lipid species in *Schwanniomyces etchellsii* grown in seawater-based (SW-YPD) or freshwater-based (YPD) medium.

Supplementary Table S1. Class representative internal standards. All lipid standards were acquired from Avanti Polar Lipids except Stearic acid, which was purchased from Merck. Stock solutions were prepared by dissolving standards at a concentration of 1 mg/mL and working solutions were diluted to 2.5 µg/ mL in MTBE.

**Compound**	**Supplier Reference**
1,3(d5)-dihexadecanoyl-2-octadecanoyl-glycerol	860902
1-palmitoyl-d31-2-oleoyl-sn-glycero-3-phosphate	860453
1-hexadecanoyl-2-(9Z-octadecenoyl)-sn-glycero-3-phospho-(1′-rac-glycerol-1′,1′,2′,3′,3′-d5)	860385
1-palmitoyl-d31-2-oleoyl-sn-glycero-3-phosphoethanolamine	860374
1-palmitoyl-d31-2-oleoyl-sn-glycero-3-phosphoinositol	860042
1-palmitoyl-d31-2-oleoyl-sn-glycero-3-[phospho-L-serine]	860403
1-hexacosanoyl(12,12,13,13-D4)-sn-glycero-3-phosphocholine	860389
25,26,26,26,27,27,27-heptadeuteriocholest-5-en-3β-ol (9Z-octadecenoate)	700185
Cholest-5-en-3β-ol(d7)	LM4100
D-erythro-sphingosine-d7-1-phosphate	860659
D-erythro-sphingosine-d7	860657
N-palmitoyl-d31-D-erythro-sphingosine	868516
N-palmitoyl-d31-D-erythro-sphingosylphosphorylcholine	868584
Stearic acid-D35	448249
1,3-Dioctadecanoyl-2-hydroxy-sn-glycerol-d5	800855

Table S2 Proteins diferentially expressed at OD_{600} 2-3

ENTRY	PROTEIN	p-value	Fold Change SW/FW
tr|W1QHM6|W1QHM6_OGAPD	Inositol-3-phosphate synthase	0,01081	4,379565035
tr|Q6BQS9|Q6BQS9_DEBHA	DEHA2E02574p	0,00408	3,411719917
tr|G8BN91|G8BN91_TETPH	Inos-1-P_synth domain-containing protein	0,01886	3,066219208
tr|G3AW91|G3AW91_CANTC	Alcohol dehydrogenase 1	0,00088	2,791875263
tr|G8BEW4|G8BEW4_CANPC	Inos-1-P_synth domain-containing protein	0,00198	2,490460951
tr|A5DHH7|A5DHH7_PICGU	Cation_ATPase_N domain-containing protein	0,00065	2,467352815
tr|A5DBS5|A5DBS5_PICGU	Semialdhyde_dh domain-containing protein	0,00331	2,351466412
tr|A0A512U616|A0A512U616_9ASCO	Uncharacterized protein	0,00423	2,161995634
tr|Q6BN36|Q6BN36_DEBHA	DEHA2F00572p	0,0061	2,094400898
tr|Q6BUN7|Q6BUN7_DEBHA	Pyruvate carboxylase	0,00047	1,958707436
tr|Q6BQH6|Q6BQH6_DEBHA	DEHA2E05148p	0,00011	1,944726969
tr|R9XBV0|R9XBV0_ASHAC	AaceriADL071Cp	0,000048036	1,878759989
tr|A0A512UN66|A0A512UN66_9ASCO	Uncharacterized protein	0,0133	1,78759855
tr|C4Y4H0|C4Y4H0_CLAL4	Pyruvate carboxylase	0,00167	1,774853253
tr|G0VIU8|G0VIU8_NAUCC	Phospho-2-dehydro-3-deoxyheptonate aldolase	0,01159	1,679072863
tr|A0A512ULD1|A0A512ULD1_9ASCO	Uncharacterized protein	0,00138	1,65027033
tr|A7TI55|A7TI55_VANPO	Dipeptidyl peptidase 3	0,01815	1,621917744
tr|N1P5E7|N1P5E7_YEASC	Bcs1p	0,0021	1,615107511
tr|Q5AJY2|Q5AJY2_CANAL	Dihydroxy-acid dehydratase	0,00115	1,612620393
tr|A0A510NY98|A0A510NY98_CANAR	Glycerol-3-phosphate dehydrogenase	0,00181	1,608966014
tr|G3B746|G3B746_CANTC	HAD-like protein	0,00094	1,578286587
tr|C4Y6H2|C4Y6H2_CLAL4	Glycerol-3-phosphate dehydrogenase	0,0101	1,565527109
tr|Q6FQJ6|Q6FQJ6_CANGA	Uncharacterized protein	0,00124	1,561066893
tr|G8Y1I2|G8Y1I2_PICSO	Pyruvate carboxylase	0,03006	1,530169497
tr|A0A2H0ZLX9|A0A2H0ZLX9_CANAR	Elongation factor Tu	0,0067	1,52882093
tr|A0A1D8PLY4|A0A1D8PLY4_CANAL	Pyruvate carboxylase	0,01829	1,512360389

ENTRY	PROTEIN	p-value	Fold Change SW/FW
tr|G8YML7|G8YML7_PICSO	Piso0_001233 protein	0,01164	0,669203114
tr|A3GEU3|A3GEU3_PICST	Fructose-1,6-bisphosphatase	0,00534	0,667319077
tr|V5EB71|V5EB71_KALBG	Succinate--CoA ligase [ADP-forming] subunit alpha, mitochondrial	0,00142	0,665932367
tr|A3GF05|A3GF05_PICST	Peroxisomal 2,4-dienoyl-CoA reductase and sorbitol utilization protein	0,01609	0,665564127
tr|G3BBU8|G3BBU8_CANTC	Phosphoglycerate mutase	0,00537	0,662193278
tr|H2B2G2|H2B2G2_KAZAF	PKS_ER domain-containing protein	0,02294	0,661325692
tr|A0A4P6XMF8|A0A4P6XMF8_9ASCO	Aldehyde dehydrogenase acceptor	0,00843	0,659231493
tr|A0A1B3B2L4|A0A1B3B2L4_9ASCO	CYP61A1	0,00879	0,657245948
tr|A0A0H5C3C5|A0A0H5C3C5_CYBJN	Glutathione peroxidase	0,00417	0,654661486
tr|G8YKZ0|G8YKZ0_PICSO	Piso0_001502 protein	0,00523	0,654304472
tr|Q6BK79|Q6BK79_DEBHA	DEHA2F24156p	0,01232	0,652860842
tr|A0A512UM49|A0A512UM49_9ASCO	Uncharacterized protein	0,00891	0,651252823
tr|Q6C638|Q6C638_YARLI	YALI0E12683p	0,01047	0,650202532
tr|A0A1V2LC73|A0A1V2LC73_CYBFA	Succinate--CoA ligase [ADP-forming] subunit beta, mitochondrial	0,00898	0,648846344
tr|H8X853|H8X853_CANO9	Sti1 protein	0,02986	0,646693452
tr|W1QIU6|W1QIU6_OGAPD	Enolase 1	0,00178	0,646519825
tr|Q6BPQ7|Q6BPQ7_DEBHA	DEHA2E11594p	0,00304	0,64266121
tr|A5DJL9|A5DJL9_PICGU	Uncharacterized protein	0,01954	0,634010898
tr|C5MAT6|C5MAT6_CANTT	Glycine cleavage system P protein	0,00077	0,631916439
tr|A5DY44|A5DY44_LODEL	Enoyl-[acyl-carrier protein] reductase 1, mitochondrial	0,00061	0,631146734
tr|H0GTZ1|H0GTZ1_SACCK	Met6p	0,04977	0,623009713
tr|M3K593|M3K593_CANMX	6-phosphogluconate dehydrogenase, decarboxylating	0,00175	0,621060118
tr|C4Y051|C4Y051_CLAL4	Uncharacterized protein	0,01916	0,620740083
tr|M3J2R2|M3J2R2_CANMX	ADP-ribosylation factor, putative	0,01006	0,619208132
tr|A0A2H1A7F9|A0A2H1A7F9_CANAR	Enolase 2	0,01045	0,618940296
tr|K0KKS6|K0KKS6_WICCF	Eukaryotic translation initiation factor 3 subunit A	0,03348	0,616763797
tr|W0T7K9|W0T7K9_KLUMD	Enolase	0,03357	0,610408555
tr|G8YE57|G8YE57_PICSO	Piso0_002113 protein	0,01355	0,598259382

ENTRY	PROTEIN	p-value	Fold Change SW/FW
tr|W1QKE6|W1QKE6_OGAPD	Heat shock protein	0,00058	0,595972338
tr|A3LST3|A3LST3_PICST	O-acetylhomoserine sulfhydrylase	0,00348	0,59558684
tr|Q6BJ01|Q6BJ01_DEBHA	DEHA2G06270p	0,00577	0,590684174
tr|W0TKF9|W0TKF9_KLUMD	5-methyltetrahydropteroyltriglutamate homocysteine methyltransferase	0,01822	0,590538899
tr|C5M841|C5M841_CANTT	AMP-binding domain-containing protein	0,00143	0,58997747
tr|G8Y5E5|G8Y5E5_PICSO	Piso0_004415 protein	0,00741	0,564150025
tr|A0A510PC49|A0A510PC49_CANAR	Uncharacterized protein	0,00043	0,551506281
tr|A0A1E3NW22|A0A1E3NW22_WICAA	Peptidyl-prolyl cis-trans isomerase	0,03888	0,551271765
tr|A3LYT9|A3LYT9_PICST	Histidine kinase osmosensor	0,01492	0,547648136
tr|C4YK87|C4YK87_CANAW	NAD-specific glutamate dehydrogenase	0,00827	0,540047349
tr|F2QVH7|F2QVH7_KOMPC	Dynamin-related GTPase protein	0,0002	0,53769754
tr|H8X9S4|H8X9S4_CANO9	Hsp70 hsp70 chaperone (Fragment)	0,01721	0,529986085
tr|G8JMR4|G8JMR4_ERECY	Serine hydroxymethyltransferase	0,00092	0,523792348
tr|A0A1E5S0H6|A0A1E5S0H6_HANUV	Fructose-bisphosphate aldolase	0,02834	0,504386222
tr|C5MJ06|C5MJ06_CANTT	Adenosylhomocysteinase	0,01236	0,464418088
tr|G8YRV7|G8YRV7_PICSO	Piso0_000914 protein	0,00052	0,440775207
tr|H8X0F9|H8X0F9_CANO9	Cdc12 septin	0,04257	0,397959292
tr|C5DBT9|C5DBT9_LACTC	KLTH0A05302p	0,02366	0,362959165
tr|Q6BMZ8|Q6BMZ8_DEBHA	DEHA2F01364p	0,00097	0,353200546
tr|A0A4P6XSF2|A0A4P6XSF2_9ASCO	Thiamine thiazole synthase	0,00012	0,108309417
tr|H8X2Z1|H8X2Z1_CANO9	Thiamine thiazole synthase	1,4973E-06	0,054639024

Overexpressed proteins in seawater medium

Overexpressed proteins in freshwater medium

Table S3 Differentially expressed proteins at DO_{600} 15-20

ENTRY	PROTEIN	p-value	Fold change (SW/FW)
tr|H8WZA6|H8WZA6_CANO9	Superoxide dismutase [Cu-Zn]	0,01943	3,143273251
tr|M3HGM4|M3HGM4_CANMX	Phosphatidate cytidylyltransferase	0,04241	2,776998148
tr|G8YH79|G8YH79_PICSO	Superoxide dismutase [Cu-Zn]	0,0037	2,177691477
tr|Q9C1R0|Q9C1R0_DEBHN	ATPase ENA1p	0,00828	2,081193958
tr|G8YFB9|G8YFB9_PICSO	Lipoyl synthase, mitochondrial	4,65E-05	1,885590826
tr|Q6CLU0|Q6CLU0_KLULA	Aldehyde dehydrogenase (NAD+) activity, mitochondria	4,71E-05	1,752738149
tr|G3AXN7|G3AXN7_CANTC	Glyceraldehyde-3-phosphate dehydrogenase	0,00788	1,648872957
tr|Q6BXY1|Q6BXY1_DEBHA	D-xilulosa reductasa	0,00011	1,631261407
tr|A3LY33|A3LY33_PICST	Peroxiredoxina, activitat peroxidasa	0,00125	1,600676009
tr|H6BJW6|H6BJW6_EXODN	Peroxidase	0,00082	1,586753881
tr|G8Y1I2|G8Y1I2_PICSO	Pyruvate carboxylase	0,0123	1,560101595
tr|A0A1E5S024|A0A1E5S024_HANUV	Adenylyl-sulfate kinase	0,00275	1,540705212
tr|C5M4D8|C5M4D8_CANTT	5-methyltetrahydropteroyltriglutamate-homocysteine S-methyltransferase	0,00969	1,513857726
tr|G8YV04|G8YV04_PICSO	Sulfate adenylyltransferase	0,0057	1,506285814
tr|C5MAT6|C5MAT6_CANTT	Glycine cleavage system P protein	0,02522	0,667757841
tr|M3K3L9|M3K3L9_CANMX	Glycosyl hydrolase, putative, cell wall	0,03412	0,667047251
tr|Q6BNB8|Q6BNB8_DEBHA	DEHA2E23078p	0,00699	0,666099754
tr|A0A2H1A7D4|A0A2H1A7D4_CANAR	Catalytic activity (?)	0,00018	0,650069101
tr|A0A512UEL1|A0A512UEL1_9ASCO	Catalase	0,00057	0,648797422
tr|C5MDM8|C5MDM8_CANTT	Histone-glutamine methyltransferase	0,00145	0,64376816
tr|A0A2Z1YIL6|A0A2Z1YIL6_9ASCO	NAD-glutamate dehydrogenase	0,00049	0,638690479
tr|K0KT50|K0KT50_WICCF	D-fructose-6-phosphate amidotransferase	0,00168	0,631491742
tr|K0KKS6|K0KKS6_WICCF	Eukaryotic translation initiation factor 3 subunit A	0,00292	0,622395456
tr|Q6BQ24|Q6BQ24_DEBHA	V-type proton ATPase subunit D in *D. fabryi* , vacuole	0,00013	0,619611895
tr|G3B6X7|G3B6X7_CANTC	ARF/SAR superfamily protein	0,0087	0,618185844
tr|A3LUS1|A3LUS1_PICST	Protein involved in nonallelic heterokaryon incompatibility	0,00639	0,609843746
tr|F2QQD5|F2QQD5_KOMPC	Peptidyl-prolyl cis-trans isomerase	0,04384	0,609426935

ENTRY	PROTEIN	p-value	Fold change (SW/FW)
tr|R7YSY0|R7YSY0_CONA1	D-fructose-6-phosphate amidotransferase	0,00073	0,605516906
tr|B9WKL5|B9WKL5_CANDC	Phosphoenolpyruvate carboxykinase (ATP)	0,02704	0,599881601
tr|G8YNK6|G8YNK6_PICSO	H/ACA ribonucleoprotein complex subunit CBF5	0,0074	0,589424858
tr|A0A1B2J5W9|A0A1B2J5W9_PICPA	ATPase activity	0,01744	0,586292561
tr|A3LPA4|A3LPA4_PICST	6-phosphogluconolactonase-like protein	0,01402	0,575056694
tr|A0A061B7J3|A0A061B7J3_CYBFA	H/ACA ribonucleoprotein complex subunit CBF5	0,02059	0,555329507
tr|W3VFL3|W3VFL3_PSEA5	Isocitrate dehydrogenase [NADP]	0,02503	0,540680927
tr|G8Y7L9|G8Y7L9_PICSO	Ribonucloprotein; 13 kDa ribonucleoprotein-associated	0,00703	0,537586243
tr|G8YS13|G8YS13_PICSO	Nucleolar protein 58	0,00162	0,528035219
tr|W1Q896|W1Q896_OGAPD	Carbamoyl-phosphate synthase arginine-specific large chain	0,00027	0,493699226
tr|Q5A8A6|Q5A8A6_CANAL	Carbamoyl-phosphate synthase (Glutamine-hydrolyzing)	3,03E-05	0,491184563
tr|G3B8S6|G3B8S6_CANTC	Ribosomal protein L35	0,0344	0,489144255
tr|A5DBA7|A5DBA7_PICGU	Serine/threonine-protein phosphatase	0,00268	0,483176248
tr|G8YRV7|G8YRV7_PICSO	Guanylate kinase	4,43E-05	0,472941403
tr|Q6BLZ6|Q6BLZ6_DEBHA	Glutathione peroxidase	0,03275	0,460365676
tr|Q6BTD9|Q6BTD9_DEBHA	Ferric-chelate reductase activity	0,0019	0,452834
tr|A0A0H5C3C5|A0A0H5C3C5_CYBJN	Glutathione peroxidase	0,01768	0,451643324
tr|B9WM32|B9WM32_CANDC	Adenylosuccinate lyase	0,04715	0,414266521
tr|R9XDR8|R9XDR8_ASHAC	Pyrimidine nucleotide transmembrane transporter activity	0,04532	0,361604601
tr|Q6BMZ9|Q6BMZ9_DEBHA	Glutaminase	0,00016	0,35335877
tr|C4YMM9|C4YMM9_CANAW	Aconitate hydratase, mitochondrial	0,02397	0,353025481
tr|I2GYC3|I2GYC3_TETBL	Nop domain-containing protein	0,03665	0,316941574
tr|S6E968|S6E968_ZYGB2	Probable pyruvate decarboxylase	0,00588	0,274189424
tr|C5DNS8|C5DNS8_LACTC	L-lactate dehydrogenase	0,00158	0,186128558
tr|G8YLA6|G8YLA6_PICSO	Polyadenylate-binding protein	0,00025	0,151147802
tr|Q6CSN8|Q6CSN8_KLULA	4-amino-5-hydroxymethyl-2-methylpyrimidine phosphate synthase	2,55E-05	0,076690702
tr|A5DLR0|A5DLR0_PICGU	Thiamine thiazole synthase	0,00209	0,056761491
tr|H8X2Z1|H8X2Z1_CANO9	Thiamine thiazole synthase	0,00018	0,028384042
tr|A0A4P6XSF2|A0A4P6XSF2_9ASCO	Thiamine thiazole synthase	0,00164	0,015636721

Proteins overexpressed in seawater medium

Proteins overexpressed in freshwater medium

Table S4 Protein with differential expression levels at OD_{600} 45-50

ENTRY	PROTEIN	p-value	Fold Change (SW/FW)
A5DHH7_PICGU	Cation_ATPase_N domain-containing protein	0,000024458	4,454745897
A5DIA7_PICGU	Aldedh domain-containing protein	0,00028	2,082062213
K1VCF0_TRIAC	Uncharacterized protein (ribosomal protein?)	0,000027958	1,806017534
A5DA06_PICGU	Amine oxidase	0,00053	0,613222692
A5DF21_PICGU	DNA-directed RNA polymerases I, II, and III subunit RPABC3	0,000084354	0,603669901
A5DKE3_PICGU	AAA domain-containing protein	0,00307	0,602288305
A5DP35_PICGU	Uncharacterized protein	0,00196	0,418515755
A5DKU4_PICGU	Glutamate dehydrogenase	0,00301	0,34472968
A3LQD6_PICST	Enolase 1	0,00289	0,254863022
A0A512U9M4_9ASCO	Uncharacterized protein	0,0021	0,170391749
F2QXG9_KOMPC	Enolase 1	0,00012	0,145442596
G3BBB4_CANTC	Uncharacteryzed protein	0,00067	0,089638533
C4Y6B1_CLAL4	Phosphoglycerate kinase	0,00107	0,031878487

Proteins overexpressed in seawater medium (rows A5DHH7_PICGU to K1VCF0_TRIAC)

Proteins overexpressed in freshwater medium (rows A5DA06_PICGU to C4Y6B1_CLAL4)

Table S5 Differential overexpressed proteins after osmotic shock

ENTRY	PROTEIN	p-value	Fold Change SW/FW
tr|A0A1Z8JNR8|A0A1Z8JNR8_PICKU	Carbamoyl-phosphate synthase arginine-specific large chain	0,00083	3,867
tr|A0A1B2JE12|A0A1B2JE12_PICPA	BA75_03733T0	0,00447	3,045
tr|G8Y2N4|G8Y2N4_PICSO	DNA mismatch repair protein	0,03777	2,753
tr|C4Y4H0|C4Y4H0_CLAL4	Pyruvate carboxylase	0,00578	2,403
tr|M3JZL6|M3JZL6_CANMX	Alpha-glucosidase	0,02312	2,402
tr|B5TYI1|B5TYI1_SCHSH	Xylitol dehydrogenase	0,00979	2,326
tr|N1P775|N1P775_YEASC	Serine/threonine-protein phosphatase	0,00787	2,251
tr|Q6FMM6|Q6FMM6_CANGA	Pyruvate carboxylase	0,02805	2,251
tr|G3AWW0|G3AWW0_CANTC	Myo-inositol-1-phosphate synthase	0,01234	2,208
tr|G3BE53|G3BE53_CANTC	Multicatalytic endopeptidase	0,02712	2,170
tr|W0TYS3|W0TYS3_DEBHA	Glycerol-3-phosphate dehydrogenase [NAD(+)]	0,00078	2,053
tr|G8YB70|G8YB70_PICSO	Transcription elongation factor SPT5	0,02121	1,975
tr|W1QLU5|W1QLU5_OGAPD	Protein URA2	0,01073	1,904
tr|A0A3M7IG63|A0A3M7IG63_HORWE	Aldedh domain-containing protein	0,01056	1,887
tr|K0KUK9|K0KUK9_WICCF	Carbamoyl-phosphate synthase / aspartate carbamoyltransferase	0,02166	1,882
tr|G8BEW4|G8BEW4_CANPC	Inos-1-P_synth domain-containing protein	0,0386	1,824
tr|G8Y9V8|G8Y9V8_PICSO	40S ribosomal protein S1	0,02069	1,809
tr|A0A1E4RXJ3|A0A1E4RXJ3_CYBJN	DNA-directed RNA polymerase subunit beta	0,0223	1,782
tr|F2QWZ5|F2QWZ5_KOMPC	Histone deacetylase complex subunit	0,01363	1,774
tr|A3GFG0|A3GFG0_PICST	40S ribosomal protein S4	0,03165	1,710
tr|Q6CTG0|Q6CTG0_KLULA	KLLA0C12991p	0,01625	1,709
tr|Q6BYD4|Q6BYD4_DEBHA	DEHA2A10384p	0,02279	1,709
tr|G8YJE1|G8YJE1_PICSO	5'-3' exoribonuclease 1	0,01128	1,696
tr|A0A0P9F8D3|A0A0P9F8D3_RHOGW	GST C-terminal domain-containing protein	0,00154	1,677
tr|Q6BM15|Q6BM15_DEBHA	Histidine biosynthesis trifunctional protein	0,00305	1,650
tr|A3LPQ2|A3LPQ2_PICST	D-3-phosphoglycerate dehydrogenase	0,01619	1,647
tr|B9WKL5|B9WKL5_CANDC	Phosphoenolpyruvate carboxykinase, putative	0,00282	1,644
tr|A0A510NY98|A0A510NY98_CANAR	Glycerol-3-phosphate dehydrogenase	0,02498	1,642

ENTRY	PROTEIN	p-value	Fold Change SW/FW
tr|A3LS42|A3LS42_PICST	Uncharacterized protein	0,01776	1,642
tr|R9XBV0|R9XBV0_ASHAC	AaceriADL071Cp	0,03599	1,642
tr|J7R3H1|J7R3H1_KAZNA	Uncharacterized protein	0,00159	1,612
tr|Q6BUN7|Q6BUN7_DEBHA	Pyruvate carboxylase	0,00036	1,610
tr|Q6C820|Q6C820_YARLI	YALI0D23463p	0,01657	1,606
tr|I2JQQ4|I2JQQ4_DEKBR	Putative carbohydrate kinase	0,00145	1,591
tr|A5DEU1|A5DEU1_PICGU	Inorganic pyrophosphatase	0,00914	1,585
tr|K0KKS6|K0KKS6_WICCF	Eukaryotic translation initiation factor 3 subunit A	0,00756	1,582
tr|C5M1X1|C5M1X1_CANTT	Uncharacterized protein	0,03704	1,570
tr|G8YQF8|G8YQF8_PICSO	Piso0_000927 protein	0,00627	1,564
tr|G8JN86|G8JN86_ERECY	Uncharacterized protein	0,02017	1,553
tr|C5MAT6|C5MAT6_CANTT	Glycine cleavage system P protein	0,01022	1,537
tr|Q6BQH6|Q6BQH6_DEBHA	DEHA2E05148p	0,01411	1,528
tr|A0A1D8PIR2|A0A1D8PIR2_CANAL	Gcn1p OS=Candida albicans	0,01503	1,518
tr|M3IR76|M3IR76_CANMX	Cytosolic leucyl-tRNA synthetase, putative	0,00366	1,505
tr|A0A0H5C7G7|A0A0H5C7G7_CYBJN	Eukaryotic translation initiation factor 2 subunit 3	0,03121	1,503
tr|Q6BKG3|Q6BKG3_DEBHA	DEHA2F22198p	0,00286	0,672
tr|Q6BMZ9|Q6BMZ9_DEBHA	DEHA2F01342p	0,00779	0,665
tr|A0A1E4S4K1|A0A1E4S4K1_CYBJN	Phosphoribosylaminoimidazolesuccinocarboxamide synthetase	0,00179	0,641
tr|C4Y951|C4Y951_CLAL4	3,4-dihydroxy-2-butanone 4-phosphate synthase	0,01586	0,635
tr|R7YKF8|R7YKF8_CONA1	Hsp98-like protein	0,03454	0,632
tr|C5M4D8|C5M4D8_CANTT	Methionine-synthesizing 5-methyltetrahydropteroyltriglutamate-homocysteine methyltransferase	0,035	0,613
tr|G8XYZ0|G8XYZ0_PICSO	Peroxidase	0,01534	0,605
tr|A0A4P6XFL1|A0A4P6XFL1_9ASCO	NADH-cytochrome b5 reductase	0,00043	0,601
tr|A5DJV7|A5DJV7_PICGU	Cytochrome c	0,01502	0,595
tr|G8YMY8|G8YMY8_PICSO	Piso0_001360 protein	0,01522	0,587
tr|Q6FVG8|Q6FVG8_CANGA	Uncharacterized protein	0,03841	0,578
tr|B5RUN6|B5RUN6_DEBHA	Glutaredoxin	0,00117	0,565
tr|M3IJZ6|M3IJZ6_CANMX	FK506-binding protein OS=Candida maltosa	0,04821	0,548

ENTRY	PROTEIN	p-value	Fold Change SW/FW
tr|W0THP2|W0THP2_KLUMD	Ubiquitin-conjugating enzyme variant MMS2	0,0311	0,546
tr|C4YCT4|C4YCT4_CANAW	Ubiquitin-related modifier 1	0,00495	0,544
tr|M3JTI3|M3JTI3_CANMX	Oleate-induced peroxisomal protein POX18	0,01149	0,541
tr|G3B6X7|G3B6X7_CANTC	ARF/SAR superfamily protein	0,00684	0,539
tr|H8X7R8|H8X7R8_CANO9	Nhp6a non-histone chromatin component	0,01576	0,538
tr|G3AYY5|G3AYY5_CANTC	Translation initiation factor SU	0,02393	0,509
tr|A0A510P5F5|A0A510P5F5_CANAR	Uncharacterized protein	0,00956	0,505
tr|G8B644|G8B644_CANPC	HTH cro/C1-type domain-containing protein	0,02788	0,498
tr|G8YF64|G8YF64_PICSO	Piso0_002487 protein	0,0002	0,480
tr|A0A4P6XDE4|A0A4P6XDE4_9ASCO	Glycolipid transfer protein GLTP	0,00026	0,473
tr|Q6CSI7|Q6CSI7_KLULA	KLLA0D00682p	0,00283	0,471
tr|A0A512UCZ2|A0A512UCZ2_9ASCO	Uncharacterized protein	0,00652	0,462
tr|A0A1B2J571|A0A1B2J571_PICPA	BA75_00168T0	0,02627	0,460
tr|Q6BND9|Q6BND9_DEBHA	Cyclin-dependent kinases regulatory subunit	0,01521	0,433
tr|Q6BL76|Q6BL76_DEBHA	DEHA2F15774p	0,00166	0,419
tr|Q59PW1|Q59PW1_CANAL	Monothiol glutaredoxin	0,0007	0,416
tr|G8YRV7|G8YRV7_PICSO	Piso0_000914 protein	0,0005	0,409
tr|A5DTR0|A5DTR0_LODEL	Peptidyl-prolyl cis-trans isomerase	0,01567	0,393
tr|M3K3L9|M3K3L9_CANMX	Glycosyl hydrolase, putative	0,00141	0,377
tr|A5DNA3|A5DNA3_PICGU	Uncharacterized protein	8,46E-05	0,376
tr|H2AVL6|H2AVL6_KAZAF	Adenylate kinase	0,02142	0,370
tr|A5DE91|A5DE91_PICGU	Uncharacterized protein	0,01035	0,369
tr|Q6BQV0|Q6BQV0_DEBHA	DEHA2E02002p	0,02401	0,369
tr|A0A0H5C3C5|A0A0H5C3C5_CYBJN	Glutathione peroxidase	0,00376	0,346
tr|Q6BHS8|Q6BHS8_DEBHA	DEHA2G16082p	0,00057	0,346
tr|A0A1V2L4N1|A0A1V2L4N1_CYBFA	Profilin	0,00741	0,346
tr|A3LNI0|A3LNI0_PICST	F1F0-ATPase subunit	0,01415	0,338
tr|A0A1E5RXI8|A0A1E5RXI8_HANUV	Guanylate kinase	0,00713	0,337
tr|A0A1E3P6Z8|A0A1E3P6Z8_WICAA	Peptidyl-prolyl cis-trans isomerase	0,00791	0,255
tr|A7TI55|A7TI55_VANPO	Dipeptidyl peptidase 3	4,91E-05	0,158
tr|C4YBA5|C4YBA5_CLAL4	Uncharacterized protein	2,68E-05	0,072

Overexpressed proteins in seawater medium

Overexpressed proteins in freshwater medium

Supplementary Table S6. List of proteins with differential expression levels at least 1.5-fold higher under more than one of the conditions considered in this work

Protein<br>Organism[1]<br>Recommended name[1]	Biological function[1]	Conditions in which it has been shown to be overexpressed
C4Y4H0_CLAL4<br>*C. lusitaniae*<br>Pyruvate carboxylase	Carbohydrate metabolic process:<br>gluconeogenesis, pyruvate metabolism	1 and 4 (seawater)
G8Y1I2_PICSO<br>*P. sorbitophila*<br>Pyruvate carboxylase	Carbohydrate metabolic process:<br>gluconeogenesis,<br>pyruvate metabolism	1 and 2 (seawater)
Q6BUN7_DEBHA<br>*D. hansenii*<br>Pyruvate carboxylase	Carbohydrate metabolic process:<br>gluconeogenesis,<br>pyruvate metabolism	1 and 4 (seawater)
A5DHH7_PICGU<br>*M. guilliermondii*<br>Q9C1R0_DEBHN<br>*D. hansenii*<br>P-type Na(+) transporter	Transmembrane transport:<br>potassium and sodium ion transport	1, 2 and 3 (seawater)
G8BEW4_CANPC<br>*C. parapsilosis*<br>Inositol 3-phosphate synthase	Lipid (phospholipid) metabolic process:<br>inositol biosynthesis	1 and 4 (seawater)
Q6BQH6_DEBHA<br>*D. hansenii*<br>DEHA2E05148p	Carbohydrate metabolic process:<br>glycerol byosynthetic process;<br>response to osmotic stress	1 and 4 (seawater)
R9XBV0_ASHAC<br>*Ashbya aceri*<br>AaceriADL071Cp	Carbohydrate metabolic process:<br>glycerol byosynthetic process;<br>response to osmotic stress	1 and 4 (seawater)
W0TYS3_DEBHA,<br>*D. hansenii*<br>A0A510NY98_CANAR<br>*Candida auris*<br>Glycerol-3-phosphate dehydrogenase [NAD(+)]	Carbohydrate metabolic process:<br>glycerol-3-phosphate catabolic process	1 and 4 (seawater)
A0A0H5C3C5_CYBJN<br>*C. jadinii*<br>Glutathione peroxidase	Cellular response to oxidative stress	1, 2 and 4 (freshwater)
G8YRV7_PICSO<br>*Pichia sorbitophila*<br>Guanylate kinase	Nucleobase-containing small molecule metabolic process;<br>Carbohydrate derivative metabolic process	1, 2 and 4 (freshwater)

H8X2Z1_CANO9<br>*C. orthopsilosis*<br>Thiamine thiazole synthase<br>A0A4P6XSF2_9ASCO<br>*M. pulcherrima*	Vitamin and sulfur compound metabolic process:<br>thiamine/thiazole biosynthetic process	1 and 2 (freshwater)
K0KKS6_WICCF<br>*Wickerhamomyces ciferrii*<br>Eukaryotic translation initiation factor 3 subunit A	Cytoplasmic translation:<br>formation of cytoplasmic translation initiation complex, translation initiation	1 and 2 (freshwater)
C5MAT6_CANTT<br>*C. tropicalis*<br>Glycine cleavage system P protein	Amino acid metabolic process:<br>glycine decarboxylation via glycine cleavage system	1 and 2 (freshwater)
G3B6X7_CANTC<br>*C. tenuis*<br>ARF/SAR superfamily protein	Transport	2 and 4 (freshwater)
M3K3L9_CANMX<br>*C. maltosa*<br>Glycosyl hydrolase, putative	Carbohydrate metabolic process;<br>cell wall organization or biogenesis	2 and 4 (freshwater)
Q6BL76_DEBHA<br>*D. hansenii*<br>DEHA2F15774p	Protein folding:<br>protein stabilization, response to unfolded protein;<br>transmembrane transport and mitochondrion organization:<br>protein import into mitochondrial matrix	2 and 4 (freshwater)
Q6BMZ9_DEBHA<br>*D. hansenii*<br>Glutaminase	Amino acid and vitamin metabolic process:<br>glutamine metabolic process, pyridoxine metabolic process.	2 and 4 (freshwater)

[1]Information obtained from *Uniprot*

Supplementary Table S7. Homologous proteins[1] in *D. hansenii*, *M. guilliermondii* and *S. cerevisiae* to proteins differentially up-expressed in seawater in the experiments described in this work.

ENTRY	HOMOLOGOUS PROTEIN IN *D. hansenii*<br>HOMOLOGOUS PROTEIN IN *M. guillermondii*<br>HOMOLOGOUS PROTEIN IN *S. cerevisiae*	Similarity (%)
W1QHM6_OGAPD<br>*O. parapolymorpha*<br>Inositol-3-phosphate synthase	Q6BJ15_DEBHA (DEHA2G05962g)<br>A5DHZ3_PICGU (PGUG_02894)<br>INO1_YEAST (Ino1)	69<br>70.5<br>72
Q6BQS9_DEBHA<br>*D. hansenii*<br>DEHA2E02574p	Q6BQS9_DEBHA (DEHA2E02574g)<br>A5DGU4_PICGU (PGUG_02495)<br>-	100<br>37.3
G3AW91_CANTC<br>*C. tenuis*<br>Alcohol dehydrogenase	Q6BH64_DEBHA (DEHA2G21032g)<br>A5DDD0_PICGU (PGUG_01281)<br>ADH1_YEAST (Adh1)	81.3<br>82.8<br>77.3
A5DHH7_PICGU<br>*M. guilliermondii*<br>P-type Na(+) transporter	Q6BIM8_DEBHA (DEHA2G09108g)<br>A5DHH7_PICGU (PGUG_02728)<br>ATN2_YEAST (Ena1)	75<br>100<br>56.3
A5DBS5_PICGU<br>*M. guilliermondii*<br>Aspartate-semialdehyde dehydrogenase	Q6BS59_DEBHA (DEHA2D11396g)<br>A5DBS5_PICGU (PGUG_00730)<br>DHAS_YEAST (Hom2)	79.1<br>100<br>75.3
A0A512U616_9ASCO<br>*Metschnikowia sp.* JCM33374<br>J domain-containing protein	Q6BKW0_DEBHA (DEHA2F18766g)<br>A5DH43_PICGU (PGUG_02594)<br>MDJ1_YEAST (Mdj1)	71.1<br>72.1<br>47.3
Q6BN36_DEBHA<br>*D. hansenii*<br>DEHA2F00572p	Q6BN36_DEBHA (DEHA2F00572g)<br>A5DL55_PICGU (PGUG_04006)<br>STL1_YEAST (Stl1)	100<br>67.1<br>57.5
H8WZA6_CANO9<br>*C. orthopsilosis*<br>Superoxide dismutase [Cu-Zn]	SODC1_DEBHA (Sod1, DEHA2G17732g)<br>-<br>-	81.8
M3HGM4_CANMX<br>*C. maltosa*<br>Phosphatidate cytidylyltransferase	Q6BNE3_DEBHA (DEHA2E22462g)<br>-<br>CDS1_YEAST (Cds1)	66.6<br><br>55.3
Q9C1R0_DEBHN<br>*D. hansenii*<br>P-type Na(+) transporter	Q6BVH9_DEBHA (DEHA2C02552g)<br>A5DHH7_PICGU (PGUG_02728)<br>ATN2_YEAST (Ena1)	99.6<br>69.9<br>56.9
A5DIA7_PICGU<br>*M. guilliermondii*	Q6BJB3_DEBHA (DEHA2G03740p)<br>A5DIA7_PICGU (PGUG_03008)	72.5<br>100

Aldehyde dehydrogenase domain-containing protein	ALDH5_YEAST (Ald5)	63.3
A0A1Z8JNR8_PICKU<br>*P. kudriavzevii*<br>Carbamoyl-phosphate synthase (ammonia)	Q6BJ15_DEBHA (DEHA2G05962g)<br>A5DHZ3_PICGU (PGUG_02894)<br>INO1_YEAST (Ino1)	69<br>70.5<br>72
G8Y2N4_PICSO<br>*P. sorbitophila*<br>DNA mismatch repair protein	-<br>A5DIU2_PICGU (PGUG_03193)<br>MSH6_YEAST (Msn6)	-<br>59.3<br>47.4
C4Y4H0_CLAL4<br>*C. lusitaniae*<br>Pyruvate carboxylase	Q6BUN7_DEBHA (DEHA2C09306g)<br>A5DKF6_PICGU (PGUG_03757)<br>PYC1_YEAST (Pyc1)	85.3<br>86.1<br>76.9
M3JZL6_CANMX<br>*C. maltosa*<br>Alpha-glucosidase	Q6BXY6_DEBHA (DEHA2A13882g)<br>A5DR30_PICGU (PGUG_05731)<br>-	68<br>66<br>-
B5TYI1_SCHSH<br>*S. shehatae*<br>Xylitol dehydrogenase	Q6BXY1_DEBHA (DEHA2A13992g)<br>A5DR25_PICGU (PGUG_05726)<br>DHSO1_YEAST (Sor1)	75.8<br>69.8<br>54.2
N1P775_YEASC<br>*S. cerevisiae*<br>Serine/threonine protein phosphatase	Q6BRM3_DEBHA (DEHA2D15290g)<br>A5DBA7_PICGU (PGUG_00562)<br>PP2A2_YEAST (Pph22)	85<br>78.3<br>100
G3AWW0_CANTC<br>*C. tenuis*<br>Inositol-3-phosphate synthase	Q6BJ15_DEBHA (DEHA2G05962g)<br>A5DHZ3_PICGU (PGUG_02894)<br>INO1_YEAST (Ino1)	80.9<br>82.2<br>70
G3BE53_CANTC<br>*C. tenuis*<br>Multicatalytic endopeptidase	Q6BK38_DEBHA (DEHA2F25146g)<br>A5DCQ4_PICGU (PGUG_01059)<br>RPN11_YEAST (Rpn11)	90.4<br>87.8<br>72.8
W0TYS3_DEBHA,<br>*D. hansenii*<br>Glycerol-3-phosphate dehydrogenase [NAD(+)]	W0TYS3_DEBHA (DEHA2E08140g)<br>A5DQE7_PICGU (PGUG_05498)<br>GPD1_YEAST (Gpd1)	100<br>79.5<br>61.3

[1]Information obtained from the Uniprot Platform

Shadowed in blue are the Ena1-like proteins.

CLUSTAL O(1.2.4) multiple sequence alignment

```
S. etchellsii          ------------------------------------------------------------
sp|P13587|ATN1_YEAST   -----------------------------MGEGTTKENNNAEFNAYHTLTAEEAAEFIGT   31
tr|Q6BVH9|Q6BVH9_DEBHA MQKEVRRSIENSKDEPHISQIYENESCEK-SKTHVNANSSEGKLAY-RLTVEQVANNFDV   58
tr|A5DHH7|A5DHH7_PICGU --------MDSSSNSSKTSKISEKRSTRRINTEDTSSGPNDGSLAY-RLTIAQVLDKYKT   51


S. etchellsii          ------------------------------------------------------------
sp|P13587|ATN1_YEAST   SLTEGLTQDEFVHRLKTVGENTLGDDTKIDYKAMVLHQVCNAMIMVLLISMIISFAMHDW   91
tr|Q6BVH9|Q6BVH9_DEBHA DVAKGLEDSRAKANLDKYGRNNLGKEEKISLTKIFAHQVFNAMILVLIISMVIALAIKDW   118
tr|A5DHH7|A5DHH7_PICGU S-RDGLSSARAKEIIDSVGPNSLGDDAKISYSKIVAHQVFNAMILVLIISMIIALAIQDW   110


S. etchellsii          ------------------------------------------------------------
sp|P13587|ATN1_YEAST   ITGGVISFVIAVNVLIGLVQEYKATKTMNSLKNLSSPNAHVIRNGKSETINSKDVVPGDI   151
tr|Q6BVH9|Q6BVH9_DEBHA ISGGVIGFVVFINIFVGFIQEYKAEKTMGSLRSLSTPSARVLRDGVESDINAEEVVPGDV   178
tr|A5DHH7|A5DHH7_PICGU ISGGVIGFVVLINIGVGFVQEVKAEKTMGSLRSLSSPTARVTRDGDDQTIPAQEVVPGDI   170


S. etchellsii          ------------------------------------------------------------
sp|P13587|ATN1_YEAST   CLVKVGDTIPADLRLIETKNFDTDESLLTGESLPVSKDANLVFGKE-EETSVGDRLNLAF   210
tr|Q6BVH9|Q6BVH9_DEBHA VCIKVGDTIPADLRLIDSMNLETDEALLTGESLPVAKCAEDVYTDSSIPVPVGDRLNMCF   238
tr|A5DHH7|A5DHH7_PICGU VHIKVGDTVPADLRLIESMNLEADEALLTGESLPVQKDENEVYADPTVPIPVGDRLNLAY   230


S. etchellsii          ------------------------------------------------------------
sp|P13587|ATN1_YEAST   SSSAVVKGRAKGIVIKTALNSEIGKIAKSLQGDSGLISRDPS---------KSWLQNTWI   261
tr|Q6BVH9|Q6BVH9_DEBHA SSSVVSKGRGTGIAVSTALNTEIGKIAKSLRSDEDALIVKVDKS---NSNFKAYIVAIAK   295
tr|A5DHH7|A5DHH7_PICGU SSSVVSKGRGTGVAIGTGLDTEIGKIASSLRGNNS-LIRKVEKSEDHSPKKREYAHAFFG   289


S. etchellsii          ------------------------------------------------------------
sp|P13587|ATN1_YEAST   STKKVTGAFLGTNVGTPLHRKLSKLAVLLFWIAVLFAIIVMASQKFDVDKRVAIYAICVA   321
tr|Q6BVH9|Q6BVH9_DEBHA STKNIICNVLGTNVGTPLQRRLAWLAIILFWVAVLFAIVVMASQEMNVNRSVAIYAICVA   355
tr|A5DHH7|A5DHH7_PICGU TIKDVVCNILGTNTGTPLQRKLSWLAIFLFWVAVVFAIVVMGSQKMHVNKEVAIYAICVA   349


S. etchellsii          ------------------------------------------------------------
sp|P13587|ATN1_YEAST   LSMIPSSLVVVLTITMSVGAAVMVSRNVIVRKLDSLEALGAVNDICSDKTGTLTQGKMLA   381
tr|Q6BVH9|Q6BVH9_DEBHA LSMIPSSLIVVLTITMAIGAQVMVTKNVIVRKLDSLEALGGINDICSDKTGTLTMGKMIA   415
tr|A5DHH7|A5DHH7_PICGU LSMIPSALIVVLTITMAVGAQVMVMKNVIVRKLDSLEALGGVNDICSDKTGTLTQGKMIA   409


S. etchellsii          ------------------------------------------------------------
sp|P13587|ATN1_YEAST   RQIWIPRFGTITISNSDDPFNPNEGNVSLIPRFSPYEYSHNEDGDVGILQNFKDRLYEKD   441
tr|Q6BVH9|Q6BVH9_DEBHA RKVWIPSTGTYAVTNSNEPFNPTVGDLSFADSSPKFIKETDEETD-----------FASK   464
tr|A5DHH7|A5DHH7_PICGU KKVWIPNIGTYSVENSNEAFNPTVGDITFADRSPYYVKETDEEID-----------FTPN   458


S. etchellsii          ------------------------------------------------------------
sp|P13587|ATN1_YEAST   LPEDIDMDLFQKWLETATLANIATVFK----DDATDCWKAHGDPTEIAIQVFATKMDLPH   497
tr|Q6BVH9|Q6BVH9_DEBHA LPEP-TPKLFEKWLETATLANIATVDQVKG-ENGTPEWEANGDATEIAINVFTTRLGLSR   522
tr|A5DHH7|A5DHH7_PICGU VPE--MPRNYRNWLYTASLANIATVNQSKDEESGELVWKAHGDATEIAIQVYASRANFGR   516


S. etchellsii          ------------------------------------------------------------
sp|P13587|ATN1_YEAST   NALTGEKSTNQSNENDQSSLSQHNEKPGSAQFEHIAEFPFDSTVKRMSSVYYNNHNETYN   557
tr|Q6BVH9|Q6BVH9_DEBHA KQM------------------------VEGNLKHIAEFPFDSSIKRMSVIYQNNQ-NSSF   557
tr|A5DHH7|A5DHH7_PICGU EQL------------------------A-ENYEHLAEFPFDSSIKRMSAVYKQNDTQTTR   551


S. etchellsii          ------------------------------------------------------------
sp|P13587|ATN1_YEAST   IYGKGAFESIISCCSSWYGKDGVK---------ITPLTDCDVETIRKNVYSLSNEGLRVL   608
tr|Q6BVH9|Q6BVH9_DEBHA IYTKGAVERVLDCCSYWYGMDGKEDMS--------PLTEADKSIIESNMNALSSEGLRVL   609
tr|A5DHH7|A5DHH7_PICGU VYTKGAVERVLACCTHWLGHSDESDKNDFDNQKPVELTKSDIEFIEENMNALSSQGLRVL   611


S. etchellsii          -----------------------------------------------------AHGDATE
sp|P13587|ATN1_YEAST   GFASKSFTKDQVNDDQLKNITSNRATAESDLVFLGLIGIYDPPRNETAGAVKKFHQAGIN   668
tr|Q6BVH9|Q6BVH9_DEBHA AFAQRSINIEKED-------ISKRESVESNLIFLGLIGIYDPPRPESAPSVKLCHKAGIN   662
tr|A5DHH7|A5DHH7_PICGU AFAVNDITGENVD-------LSERENVEKNLTFQGLIGIYDPPRTETAHSVKLCHKAGVN   664
                                                                            *  . :
```

```
S. etchellsii          ----IAIQVYASRAIAQEVGIIPRRINTEDTSSGPN--DGSLAYRVTRDGDDQTIPAQEV
sp|P13587|ATN1_YEAST   VHMLTGDFVGTAKAIAQEVGILPTNLYHYSQEIVDSMVMTGSQFDGLSEEEVDDLPVLPL   728
tr|Q6BVH9|Q6BVH9_DEBHA VHMLTGDHHGTARAIAQEVGILPSNLYHYTEEVVKSMVMTANDFDALSNDEIDNLPVLPL   722
tr|A5DHH7|A5DHH7_PICGU VHMLTGDHPGTAKAIAQEVGIIPRNLYHYSEEVVKAMCMTASEFDALSDADIDNLPVLPL   724
                            .    :::********:* .:     .        .  :    : : : :*.  :

S. etchellsii          -----VPGDIVHIKNLYHYSEEVVKFVAMTGDGVNDSPSLK----------------KDA
sp|P13587|ATN1_YEAST   VIARCSPQTKVRM---IEALHRRKKFCTMTGDGVNDSPSLKMANVGIAMGINGSDVSKEA   785
tr|Q6BVH9|Q6BVH9_DEBHA VIARCAPQTKVRM---IEALHRRGKFVAMTGDGVNDSPSLKKADVGIAMGLNGSDVAKDA   779
tr|A5DHH7|A5DHH7_PICGU VIARCAPQTKVRM---IEALHRREKFVAMTGDGVNDSPSLKKADVGIAMGLNGSDVAKDA   781
                             *   *::    .  ..  ** :*************                *:*

S. etchellsii          SDIVLTDDNFASILNAIEEGRRHIAEFP---FDSSIKRKLDSLEALGGINDICSD-----
sp|P13587|ATN1_YEAST   SDIVLSDDNFASILNAVEEGRRMTDNIQKFVLQLLAENVAQALYLIIGLVFRDENGKSVF   845
tr|Q6BVH9|Q6BVH9_DEBHA SDIVLTDDNFASILNAIEEGRRMSSNIQKFVLQLLAENVAQALYLMIGLAFMDKDGFSVF   839
tr|A5DHH7|A5DHH7_PICGU SDIVLTDDNFASILNAIEEGRRMSANIQKFVLQLLAENVAQAFYLMIGLAFMDKSGYSVF   841
                       *****:*********:*****    ::    ::   :. :::  :  *:     ..

S. etchellsii          -----------KKADVGIAMGLNGSDVAK-------------------------------
sp|P13587|ATN1_YEAST   PLSPVEVLWIIVVTSCFPAMGLGLEKAAPDLMDRPPHDSEVGIFTWEVIIDTFAYGIIMT   905
tr|Q6BVH9|Q6BVH9_DEBHA PLAPVEVLWIIVVTSCFPAMGLGQEKANDDILEQPPNA---TIFTWEVIIDMIAYGFWMA   896
tr|A5DHH7|A5DHH7_PICGU PLSPVEVLWILVVTSCFPAMGLGQEKASEDILDQPPNN---TIFTYEVIGDMVAYGFWMS   898
                                    :.   ****. ...

S. etchellsii          ------------------------------------------------------------
sp|P13587|ATN1_YEAST   GSCMASFTGSLYGINSGRLGHDCDGTYN--SSCRDVYRSRSAAFATMTWCALILAWEVVD   963
tr|Q6BVH9|Q6BVH9_DEBHA CCCLTCFVLIVFAVGDGNLGSNCNDSGG--DSCNLVFRGRSGAFATFTWCALLLAWECIH   954
tr|A5DHH7|A5DHH7_PICGU CCCLLCFVVIVFGKGDGYLGENCNSTSADKHVCELVFKGRSASFANMTWCALILAWECIH   958


S. etchellsii          ------------------------------------------------------------
sp|P13587|ATN1_YEAST   MRRSFFRMHPDTDSPV-KEFFRSIWGNQFLFWSIIFGFVSAFPVVYIPVINDKVFLHKPI   1022
tr|Q6BVH9|Q6BVH9_DEBHA MRYSFFNMRPESEISRGKQLAIDLWDNQFLFWSIIGGFLSVFPVVYIPVINDIVFLHDPI   1014
tr|A5DHH7|A5DHH7_PICGU PVNSLFYMRQDTDNPWWKQTAIDLWDNQFLFWSIIGGFVSVFPVVYIPVINDKVFLHGPI   1018


S. etchellsii          ------------------------------------------------------------
sp|P13587|ATN1_YEAST   GAEWGLAIAFTIAFWIGAELYKCGKRRYFKTQ--RAHNPENDLESNNKRDPFEAYSTSTT   1080
tr|Q6BVH9|Q6BVH9_DEBHA GYEWGLAVGFTLIFLLGAELWKWFKRIYFRKST--IKNPEYDLEKNDPFMKYSSFSKSNT   1072
tr|A5DHH7|A5DHH7_PICGU GYEWGVAIGCSVLFLLGAEGWKWIKRIYKRKKAKKAKNPEHDLERNDPFQKYASFSRSNT   1078


S. etchellsii          -----------
sp|P13587|ATN1_YEAST   IHTEVNIGIKQ    1091
tr|Q6BVH9|Q6BVH9_DEBHA MEVS-------    1076
tr|A5DHH7|A5DHH7_PICGU MDKTDYVV---    1086
```

Supplementary Figure S1. Sequence alignment of Ena1 homologs in *S. cerevisiae* and in several non-*Saccharomyces* yeasts with the sequence obtained from the proteomic analysis carried out in *S. etchellsii*. Similar amino acids are indicated by "." or ":" and identical amino acids by "*".*S. etchellsii* refers to the protein found in this study, YEAST to *S. cerevisiae*, DEBHA to *D. hansenii* and PICGU to *M. guilliermondii*.

Supplementary Figure S2

A

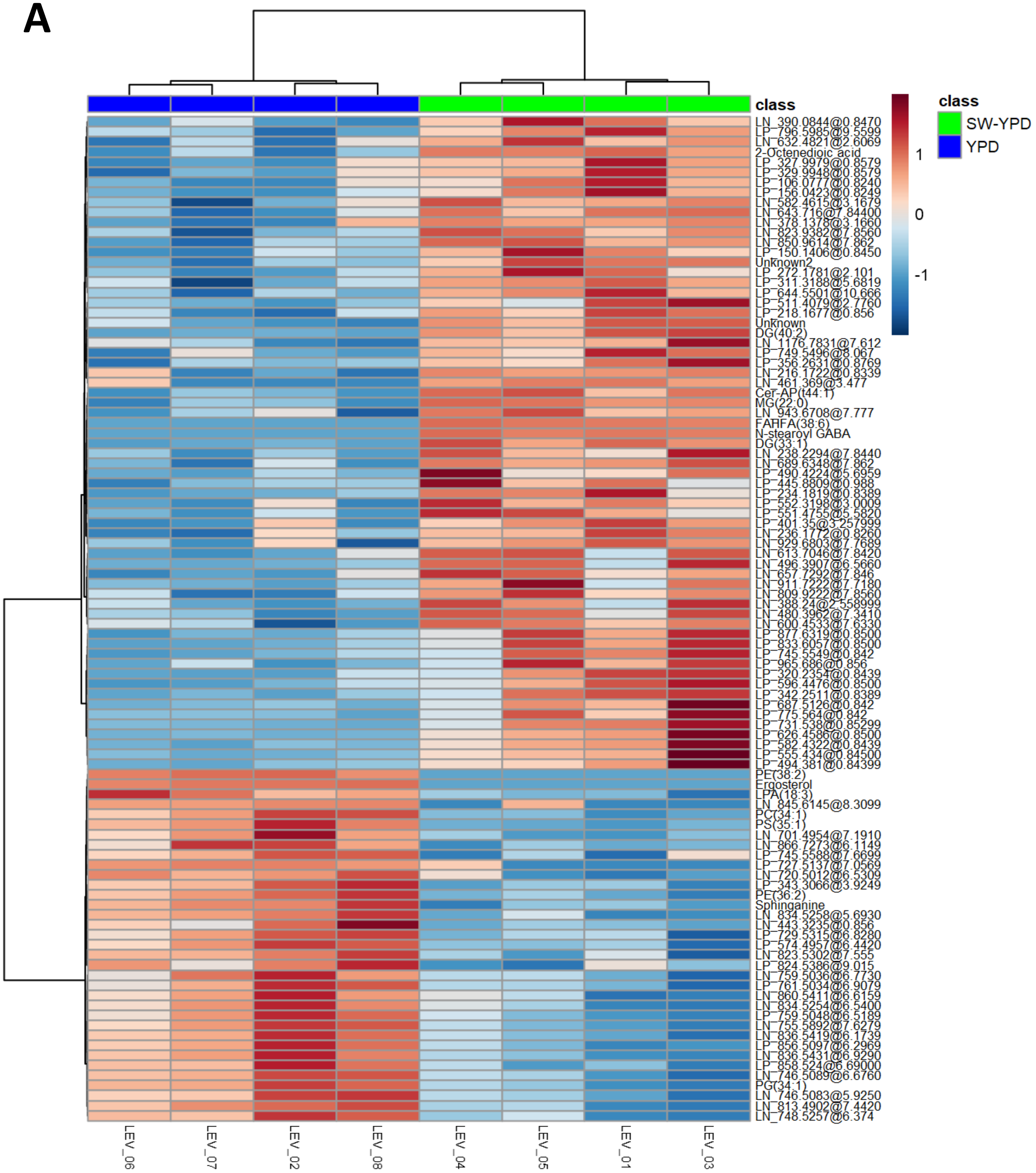

Supplementary Figure S2

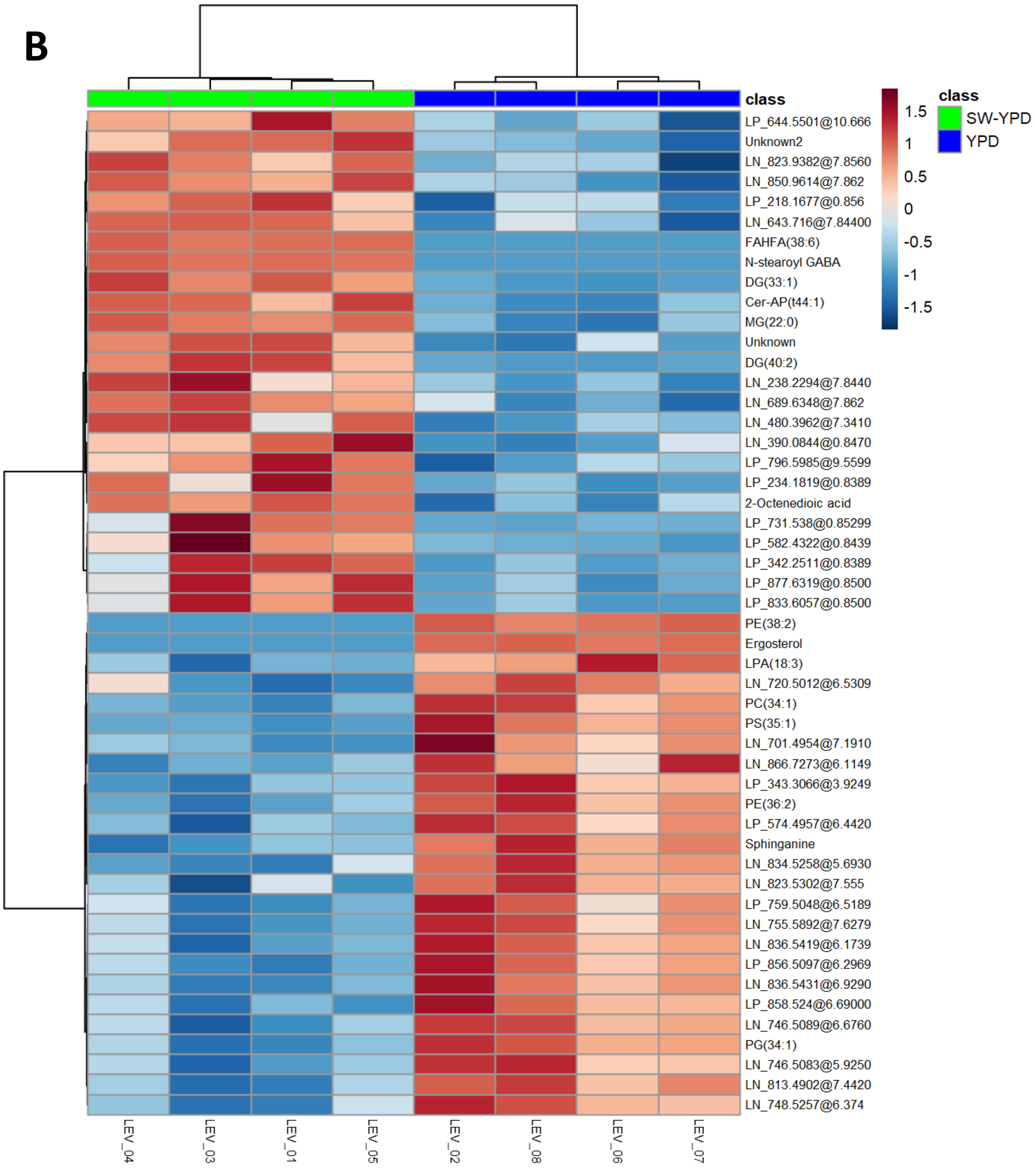

Supplementary Figure S2. Heatmaps showing the relative abundance of lipid species with the most significant differences between *S. etchellsii* cells grown in SW-YPD and YPD. The figure includes heatmaps for the top 100 (panel A) and top 50 (panel B) most differentially abundant lipid compounds across the two growth conditions according to T-test.

Supplementary Figure S3

A

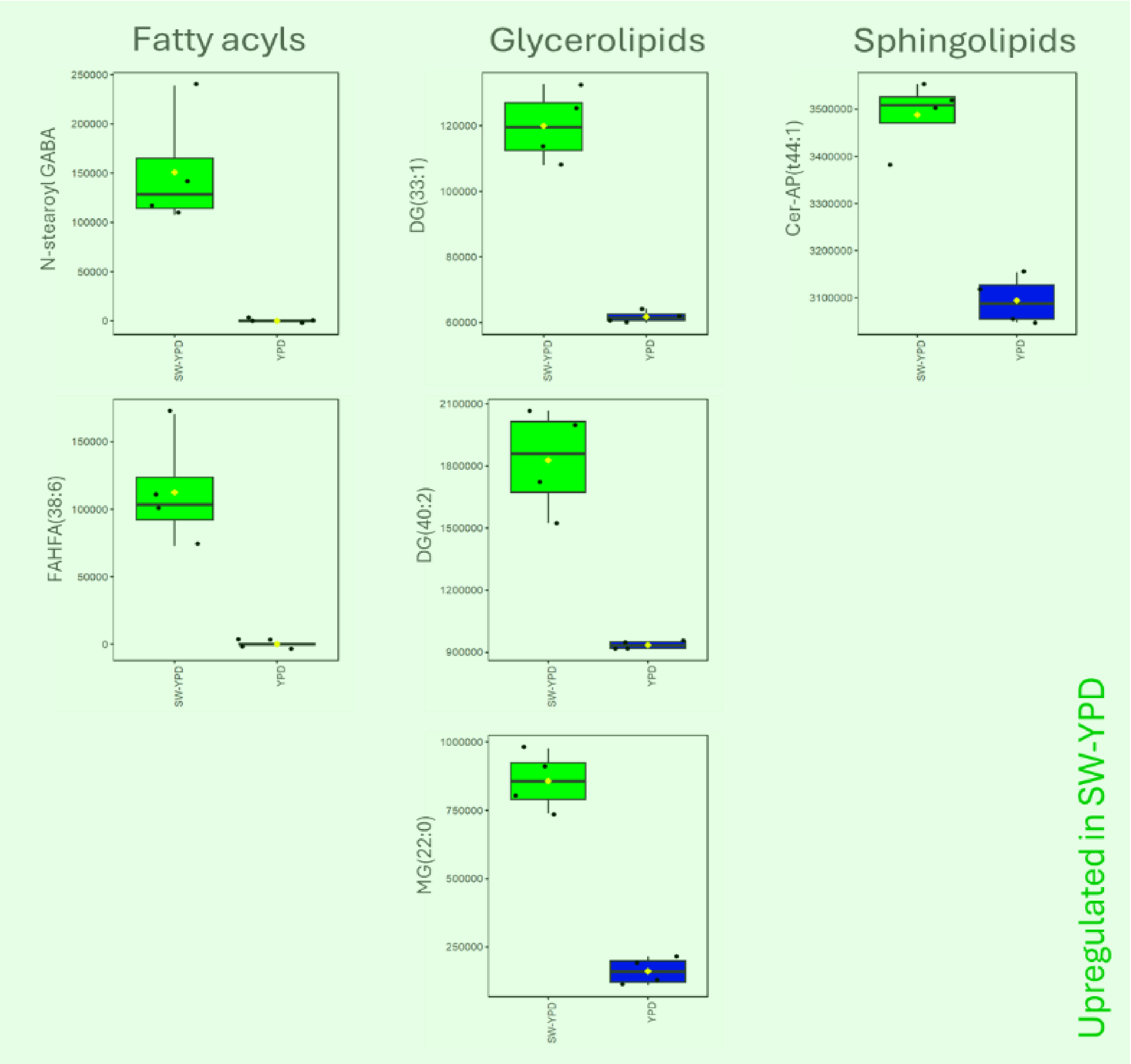

Supplementary Figure S3

B

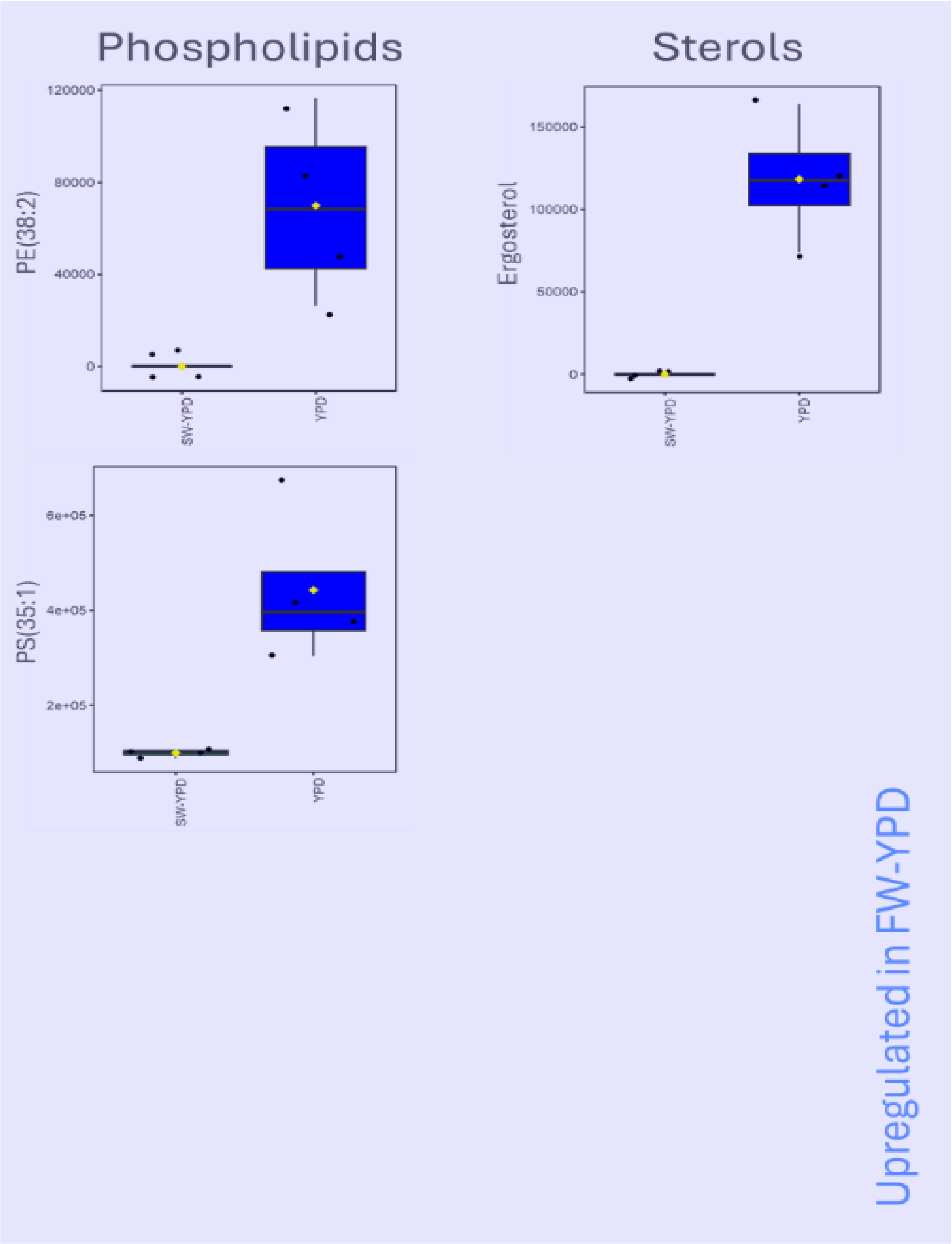

Supplementary Figure S3. Differential lipid species in *Schwanniomyces etchellsii* grown in seawater-based (SW-YPD, panel A) or freshwater-based (YPD, panel B) medium. Boxplots of the 9 lipid compounds found to differ significantly between conditions in the untargeted lipidomic analysis (T-test with FDR adjusted p-value < 0.05). Lipids shown in green were more abundant in SW-YPD (including fatty acyls, glycerolipids, and one sphingolipid), whereas those in blue were more abundant in YPD (phospholipids and sterols, including ergosterol).
